# Early palliative care for patients with oral cancer in Sri Lanka: A non-randomized controlled trial

**DOI:** 10.1371/journal.pgph.0005985

**Published:** 2026-03-09

**Authors:** Nadisha Ratnasekera, Irosha Perera, Sumeth Perera, Jenny Lau, Camilla Zimmermann, Pushpakumara Kandapola Arachchige

**Affiliations:** 1 Postgraduate Institute of Medicine, University of Colombo, Colombo, Sri Lanka; 2 Department of Supportive Care, Princess Margaret Cancer Centre, University Health Network, Toronto, Ontario, Canada; 3 Preventive Oral Health Unit, National Dental Hospital (Teaching) Sri Lanka, Colombo, Sri Lanka; 4 Department of Biochemistry, Faculty of Medicine, Sabaragamuwa University of Sri Lanka, Balangoda, Sri Lanka; 5 Princess Margaret Cancer Centre Research Institute, University Health Network, Toronto, Ontario, Canada; 6 Division of Palliative Care, Department of Family and Community Medicine, University of Toronto, Toronto, Ontario, Canada; 7 Department of Psychiatry, Faculty of Medicine, University of Toronto, Toronto, Ontario, Canada; 8 Division of Palliative Medicine, Faculty of Medicine, University of Toronto, Toronto, Ontario, Canada; 9 Library and Information Services, University Health Network, Toronto, Ontario, Canada; 10 National Cancer Institute, Maharagama-Apeksha Hospital, Maharagama, Sri Lanka; PLOS: Public Library of Science, UNITED STATES OF AMERICA

## Abstract

Oral cancer is prevalent among Sri Lankan men and substantially compromises quality of life (QOL). Limited evidence exists on early palliative care in low- and middle-income countries. We assessed the effectiveness of an early palliative care intervention in improving psychological distress and QOL among patients with oral cancer in Sri Lanka. We conducted a non-randomized controlled trial with group allocation based on patients’ residence. The study is registered as NCT06726317. We recruited from four tertiary care units providing oral cancer treatment in Sri Lanka: the oral and maxillofacial wards at the National Dental Hospital, Colombo (Teaching); Colombo South Teaching Hospital, Karapitiya; and the onco-surgery wards at the National Cancer Institute, Maharagama (Apeksha Hospital). The eligible participants were patients with oral cancer who had received their definitive diagnosis, reported psychological distress (score ≥4 on the Sinhala version of the Distress Thermometer), were awaiting surgery as the first treatment modality, and were married with at least one family caregiver able to communicate in Sinhalese. Exclusion criteria included recurrent oral cancer, a formal psychiatric diagnosis, or prior receipt of early palliative care. Participants were assigned to the intervention (n = 55) or control group (n = 55) based on the availability of an accessible Public Health Nursing Officer. The intervention group received an early palliative care package consisting of three structured sessions. The first session was delivered by the principal investigator (a trained dental surgeon and public health professional) during the hospital stay before surgery. The second and third sessions were provided by trained Public Health Nursing Officers at participants’ homes, one and three weeks after discharge. Each session lasted about two hours and included information provision, management of acute and functional issues, nutritional care, psychological support, mindfulness therapy, and coordination of financial assistance. The control group received standard care within the health system, which included ad hoc symptom management on request without a structured palliative care component. The effectiveness was assessed using the Sinhala version of the Distress Thermometer for psychological distress and the EORTC QLQ-C30 with the H&N35 module for QOL at baseline (T0), post-intervention (T1), one month (T2), and three months (T3). All 110 eligible participants completed the intervention and follow-up (response rate 100%). At baseline, there were no significant differences between groups in distress (p = 0.7) or QOL. Over time, the intervention group showed greater reductions in distress (mean difference = –2.26; 95% CI: –3.35 to –1.07; p = 0.0001). After adjusting for baseline scores and potential confounders, the intervention group reported higher Global Health Status (17.1; 95% CI: 6.9–27.3; p = 0.001) and Functional Status (23.6; 95% CI: 5.7–41.5; p = 0.01), as well as lower Symptom Status (23.2; 95% CI: 5.6–40.8; p = 0.01) and H&N35 symptom burden (16.8; 95% CI: 6.8–26.8; p = 0.001) compared with controls. We conclude that the early palliative care intervention showed promising effectiveness in reducing psychological distress and improving quality of life among patients with oral cancer in Sri Lanka. Public Health Nursing Officers may play a key role in linking tertiary and community care to enhance patient well-being. Larger studies across diverse populations are needed to confirm these findings.

## Introduction

Oral cancer is one of the most common cancers in the world [1–3], and the most common cancer in Sri Lankan men [4], with a profound effect on the quality of life of patients and their families. After a diagnosis of oral cancer, patients experience not only disturbance of physical appearance and basic human functions such as eating, speech, and breathing [5–7], but also emotional, social, and spiritual problems [8,9]. Psychological distress can adversely affect compliance with medical care, recovery from illness, adjustment to life after treatment, and survival [10–14].

The World Health Organization defines palliative care as, “an approach that improves the quality of life of patients and their families who are facing problems associated with life-threatening illness. It prevents and relieves suffering through the early identification, correct assessment, and treatment of pain and other problems” [15]. In high-income countries, randomized controlled trials have shown that early palliative care for patients with advanced cancer improves the quality of life, mood, satisfaction with care, and symptom control [16–19]; this evidence has led to recommendations by major cancer organizations that early palliative care be enacted for all patients with advanced cancer [20]. In addition, the National Comprehensive Cancer Network (NCCN) recommends routine screening for distress among all patients with cancer and considers psychological distress as the sixth vital sign of cancer care [14]. However, neither distress screening nor integration of palliative care are routinely available in low- and middle-income countries (LMICs), and there has been scant prospective research on the impact of early palliative care in these countries [21].

In the Sri Lankan health system, inpatient cancer care is provided mainly through the public sector, which provides universal access to cancer treatment, and outpatient cancer care is delivered through both the public and private sectors [22]. Sri Lanka has a national strategic framework for palliative care development (from 2018 to 2023), which includes pain and symptom management; psychological, emotional, social, and spiritual support; and support of family and caregiver coping during the patient’s illness and bereavement period [23]. Palliative care is mainly delivered by oncologists and consultant psychiatrists in the 25 cancer centres across Sri Lanka [24]. The only specialized palliative care clinic is at the National Cancer Institute Sri Lanka - Apeksha Hospital, headed by a consultant oncologist [24]. In 2018, a postgraduate diploma program in Palliative Medicine was introduced to address the need for professionals in palliative care in Sri Lanka [24]. Despite these advances, palliative care for patients with cancer is provided only at the end of life and there is no special team assigned to provide palliative care in Sri Lanka [24,25].

The aim of this study was to determine the effectiveness of an early palliative care intervention in improving psychological distress and quality of life among patients with oral cancer in Sri Lanka.

## Methods

This study was reported in accordance with the TREND statement (S1 Appendix) [26]. This study was registered in ClinicalTrials.gov as number NCT06726317 https://register.clinicaltrials.gov/prs/beta/studies/S000F3DX00000052/recordSummary. The study was originally designed and conducted as a quasi-experimental evaluation embedded within routine clinical services. At the time of study initiation, it was understood that such quasi-experimental designs did not require prospective clinical trial registration. However, upon later review of international reporting standards, we recognized that registration would strengthen transparency and methodological clarity. Therefore, the trial was registered retrospectively. No changes were made to the study design, outcomes, or analysis plan after participant enrolment began.

### Study design and participants

This was a multi-center non-randomized controlled trial where participants were allocated to intervention or control groups according to whether their area of residence had access to Public Health Nursing Officers.. The study took place at four tertiary care units providing oral cancer treatment in Sri Lanka: the oral and maxillofacial wards at the National Dental Hospital, Colombo (Teaching); the oral and maxillofacial wards at Colombo South (Teaching) Hospital, Karapitiya; and the onco-surgery wards at the National Cancer Institute Maharagama (Apeksha Hospital) from 29, July 2019–30, May 2020. Inclusion criteria were: definitive diagnosis communicated to the patient; presence of psychological distress (score of ≥4 after screening with the Sinhala version of the Distress Thermometer [27]); awaiting surgery as the first treatment modality; ability to communicate and read in Sinhalese; married with children with at least one family caregiver (spouse or child) serving as the primary caregiver [28]. Exclusion criteria were: recurrent oral cancer; a formal psychiatric diagnosis; and receiving or having received any early palliative care intervention. The study was approved by the Ethics Board, Faculty of Medicine, University of Colombo, Sri Lanka (registration number EC- 18–097) and with the 1964 Helsinki Declaration and its later amendments or comparable ethical standards.

### Study procedures

The PI made regular visits to the Oral and Maxillofacial Surgery wards and screened patients admitted for surgery using the ward admission lists. All patients who fulfilled the eligibility criteria were approached for recruitment. The PI met patients at the ward when they were admitted for the surgery. The eligible patients were introduced to the study by the PI, and the information sheet was given. Enough time was given for any clarification. Once the participant was confident about the study the consent form was given before study commencement. Informed written consent was obtained from all individual participants included in the study.

### Group assignment

Participants were divided into two groups based on the availability of an accessible Public Health Nursing Officer. The Public Health Nursing Officer, a recently established healthcare category in Sri Lanka, attached to Healthy Lifestyle Centres in selected base hospitals [29]. They provide both hospital and community-based services, with responsibilities spanning non-communicable disease prevention, palliative and geriatric care, and special services such as disaster management. Within palliative care, their role focuses on alleviating symptoms and improving the quality of life of patients with chronic illnesses and their families [29]. An “accessible PHNO” was defined as one who could reach the residence of an eligible patient within a maximum of 1.5 hours by personal or public transport. Eligible patients with oral cancer residing in areas covered by an accessible PHNO were allocated to the intervention group, while those living in areas without one were assigned to the control group.

### Intervention group

The early palliative care intervention package (Table 1 and S2 Appendix) for patients with oral cancer was developed following the UK Medical Research Council guidelines for complex interventions [30]. It comprised six components: (1) provision of information, (2) management of acute and functional issues, (3) nutritional care, (4) psychological support, (5) mindfulness therapy, and (6) coordination of financial assistance. The content was formulated through triangulation of multiple data sources, including a literature review, a case–control study, in-depth interviews with oncology and palliative care experts, key informant interviews with patients and caregivers, and observational studies conducted at the National Cancer Institute, Sri Lanka. The final version was refined through a nominal group discussion with experts in oncology, oro-maxillo-facial surgery, psychiatry, sociology, and psychology [28]. Details of the intervention and the TIDieR checklist are provided in S2 and S3 Appendices.

**Table 1:** Comparison of the early palliative care intervention package and standard care.

Description	Standard Care	Standard Care + Early palliative care
Overall care aspects		
Care in the ward (Once the patient got admitted to the ward for the surgery)	Routine oncology care only	Routine oncology care plus the PI visited to deliver the first session of the intervention package
At the discharge	Routine oncology care. Based on the oncologist’s decision some patients received a consultation with the dietician.	Routine oncology care plus the patient was provided with an information-providing booklet and a dietary plan after the consultation with the dietician in the hospital. The patient was connected to the Public Health Nursing Officer of the area
Home care	No home care	Public Health Nursing Officer visited the patient’s residence twice with a two-week gap and delivered the last two sessions of the intervention
Components of the intervention		
Providing information (The information included the status of the condition, about the surgery, the life after surgery)	Routine method - talking to the medical/dental officers and nursing officers, during clinic dates and ward rounds	Routine method An information-providing booklet and a video clip were given In-person discussion with the PI and Public Health Nursing Officer
Addressing acute and functional issues	Routine method - seeking help from the medical/dental officers and nursing officers, during clinic dates and ward rounds	Routine method Direct access to the PI by phone and to the Public Health Nursing Officer by phone and when required in-person visists Hassle free referrals for pain and other acute issues management
Nutritional care	Routine method – ad-hoc. Referral to the dietician depends on the oncologist’s decision. If the patient complained of some difficulty in taking food it was addressed.	Routine method A consultation with the dietician (after the surgery before the discharge from the ward). Provision of a customized diet plan Follow-up on the compliance to the diet plan and support through the Public Health Nursing Officer
Psychological support	No special support	Problem-solving counseling and follow up care
Mindfulness therapy	No therapy	Mindfulness based therapy and follow up care by the PI and the Public Health Nursing Officer.
Coordination of the financial allowance	Contact with social workers at District Secretariat, depends only on patients’ initiative. Therefore, the process is slow and uncertain.	The PI and the Public Health Nursing Officer reached out to the social worker at the relevant District Secretariat office and made sure the financial allowance was received without delay or any hassle.

The intervention was delivered in three sessions. The first session was conducted by the PI at the hospital following patient consent, prior to surgery. Subsequently, the relevant Public Health Nursing Officer in the patient’s area carried out the second and third sessions at the patient’s home, one and three weeks after hospital discharge, respectively. Each session lasted approximately two hours.

To ensure consistent delivery, Public Health Nursing Officers who implemented the intervention received structured training and a handbook with guidance [28,29]. The training program, developed with input from multidisciplinary experts, consisted of two parts: a two-day, in-person session for all Public Health Nursing Officers in Sri Lanka, followed by an online module for those selected to deliver the intervention [31]. Training effectiveness was assessed using the Kirkpatrick Training Evaluation Model [32]. A detailed intervention handbook was provided to guide Public Health Nursing Officers in the delivery process.

Intervention fidelity was maintained through regular monitoring and follow-up. The PI contacted each Public Health Nursing Officer three times during the intervention phase to provide support and ensure fidelity to the protocol. The first contact occurred when the patient was referred to the PHNO to deliver the intervention. Subsequent contacts took place before and after each home-based session to review progress, address challenges, and provide corrective guidance as needed. These communications helped ensure consistent delivery of the intervention and allowed the PI to coordinate patient follow-up and assess adherence. Patient follow-up visits were scheduled at convenient times, preferably on clinic days, and reminders were given twice before each appointment to enhance compliance. S2 Appendix gives a comprehensive detail about the intervention and S3 Appendix provides the TIDieR checklist.

### Control group

The control group received standard care available within the existing health system. The Principal Investigator and Public Health Nursing Officers were not involved in providing care to control participants. Access to palliative care services was limited to instances where patients were referred by their treating oncologist, usually on an ad hoc basis. These services primarily focused on managing physical symptoms, without a structured or continuous palliative care component. Table 1 shows the comparison of the early palliative care intervention and the standard care received by the intervention and control groups, respectively.

### Outcome measures

Data were collected by the PI and the Public Health Nursing Officers. The outcome measures were collected at four-time points: at enrollment just before the intervention was delivered (T_0_) (by the PI at the tertiary care hospital); immediately after delivering the final session of the intervention package during the same encounter (T_1_) (by the Public Health Nursing Officer at the patient’s residence); and 1 month (T_2_) and 3 months (T_3_) after the intervention (by the PI at the tertiary care hospital during clinic visits).

The primary outcomes were the level of psychological distress and quality of life of the patient. The level of psychological distress was measured through the mean scores of the Sinhala version of the Distress Thermometer [27]. The Distress Thermometer is 1 item, visual analogue screening tool ranging from 0 (no distress) to 10 (extreme distress), with higher scores indicating greater psychological distress [33]. The Distress Thermometer was translated into the Sinhala language (the primary language in Sri Lanka) and cross-culturally adapted to Sri Lanka [27].

Quality of life was measured using the Sinhala version of the EORTC QLQ 30 and H&N 35 [34]. These tools were translated and validated to Sri Lanka through a large-scale multicentre study [34]. The quality of life subdimension scores were calculated as per the EORTC QLQ-Q30 Scoring Manual [35]. Accordingly, a high score in Global Health Status (GHS) denoted a high quality of life, a high score for Functional Scale (FS) meant healthy level of functioning, a high score in Symptom Scale (SS) represented high level of problems and a high score in H&N-35 module also meant high level of problems.

Additionally, process evaluation indicators measured the reception of the intervention package within and between the groups. These were collected by PI at the final follow up visit. The results are presented in S4 Appendix.

### Statistical methods

The sample size was calculated using a minimally important difference (MID) of 2 units in quality of life between the two groups [36], and a standard deviation of 4, giving a standardized effect size of 0.5, with a significance level (α) of 0.10 and power (1 – β) of 80%. Using these parameters and a design factor of 6.2 from standard sample size tables, the required sample size was estimated to be 50 participants per group. After accounting for a potential non-response rate of 5–10%, we set a final target sample size of 55 per group (110 in total).

All analyses were performed by intention to treat, with analysis within the groups to which patients had originally been assigned, regardless of whether or not they actually received their intended intervention. SPSS version 21 software package was used for data analysis. The baseline sociodemographic data, socioeconomic data, and other selected characteristics were compared between the intervention group and the control group.

Distress Thermometer scores and quality of life subscale scores were compared between the intervention and control groups at each time point, and within each group over time to assess changes following the intervention. The EORTC QLQ 30-H&N 35 subscale scores were calculated as per the EORTC QLQ-Q30 Scoring Manual [35]. The scores distributions were assessed for normality. Since the data was in a skewed distribution, the Mann-Whitney U test was used. However, descriptive statistics are presented as mean (SD) to facilitate interpretability. Linear regression was carried out to control for the potential confounders and assess the impact of the novel intervention on psychological distress and quality of lifeat the final follow-up time point (T3).

## Results

**Fig 1:**
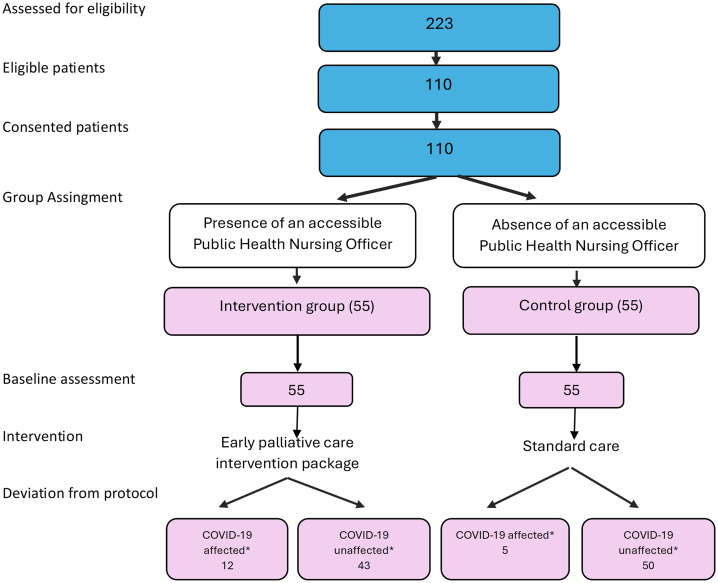
Patients progress through the study – flow chart. * COVID-19 affected – * COVID-19–affected participants – Participants whose scheduled timing for the delivery of the intervention sessions, standard care, or follow-up data collection was delayed or rescheduled due to restrictions or disruptions related to the COVID-19 pandemic (e.g., unavailability of Public Health Nursing Officers for home visits, or inability of participants to attend hospital clinic visits because of lockdowns or travel restrictions). * COVID-19 unaffected participants ** COVID-19–unaffected participants – Participants whose intervention delivery, standard care, and data collection occurred according to the planned schedule and were not delayed or disrupted by the COVID-19 pandemic.

Of 223 patients who were screened, 110 were eligible and provided follow-up data for all time points, with a response rate of 100%. Although there was no loss to follow-up, 12 participants of the intervention group and 5 in the control group had delayed delivery of the intervention and delayed data collection due to the COVID-19 pandemic (Fig 1).

As per the planned intention-to-treat analysis, all participants (whether or not the intervention and data collection were affected due to COVID-19) were analyzed within the study group that they were originally assigned.

### Sample characteristics

Table 2 shows the socio-demographic, socio-economic and medical characteristics of the study participants. A total of 110 patients with oral cancer participated in the study, with 55 in each group. The mean age of participants was 56.7 years (SD = 10.6), and the majority were male (77.3%). Most participants were of Sinhalese ethnicity (81.8%), followed by Tamil (10.9%) and Moor or other ethnic groups (7.3%). In terms of education, 57.3% had completed secondary education, while 11.8% had no formal schooling. About half of the participants (50.9%) were unemployed. The largest proportions of patients were treated at the National Dental Hospital (32.7%) and Karapitiya Teaching Hospital (30.9%). Most patients (70%) were diagnosed with late-stage disease. No significant differences were observed between the intervention and control groups in any of these baseline characteristics.

**Table 2:** Comparison of the selected characteristics of the intervention group and control group.

Characteristic	IG^a^ (n = 55) No. (%)	CG^b^ (n = 55) No. (%)	Total (n = 110) No. (%)	P value*
**Selected socio-demographic characteristics**				
**Age (Years)**	**Median = 55 (Range = 47–65)**	**Median = 61, (Range = 45–68)**	**Median = 58 (Range = 38–76)**	0.41
30-40	7 (12.7)	4 (7.3)	11 (10)	
41-59	27 (49.1)	24 (43.6)	51 (46.4)	
60-80	21 (38.2)	27 (49.1)	48 (43.6)	
**Sex**				
Male	43 (78.2)	42 (76.4)	85 (77.3)	0.82
Female	12 (21.8)	13 (23.6)	25 (22.7)	
**Ethnicity**				
Sinhala	47 (85.4)	43 (78.2)	90 (81.8)	0.47
Tamils	4 (7.3)	8 (14.5)	12 (10.9)	
Moors and others	4 (7.3)	4 (7.3)	8 (7.3)	
**Selected socio-economic characteristics**				
**Level of education**				
No schooling	4 (7.3)	9 (16.4)	13 (11.8)	0.34
Primary complete	18 (32.7)	16 (29.1)	34 (3.1)	
Secondary complete	33 (60.0)	30 (54.5)	63 (57.3)	
**Occupation category**				
Unemployed/ Retired	23 (41.8)	33 (60.0)	56 (50.9)	0.073
Employed				
Skilled^w^ and unskilled^x^	27 (49.1)	21 (38.2)	48 (87.3)	
Professional and clerical	5 (9.1)	1 (1.8)	6 (10.9)	
**Selected medical characteristics**				
**Treated tertiary care institute**				
Apeksha Hospital	15 (27.3)	10 (18.2)	25 (23.7)	0.84
National Dental Hospital	13 (23.6)	23 (39.9)	36 (32.7)	
Karapitiya Teaching Hospital	17 (30.9)	17 (30.9)	34 (30.9)	
Colombo South Teaching Hospital	5 (9.1)	10 (14.9)	15 (13.6)	
**Stage of the disease**				
Early^y^	17 (30.9)	16 (29.1)	33 (30.0)	^*^ 0.24
Late^z^	38 (69.1)	39 (70.1)	77 (70.0)	

(a **=** Intervention group**,** b = Control group, ^*^ = Chi Square, w- labourers who have undergone a short training, x- labourers who have not undergone any training, y = Presence of the local lesion only, z = the lymph nodes are involved).

### Level of psychological distress

**Fig 2:**
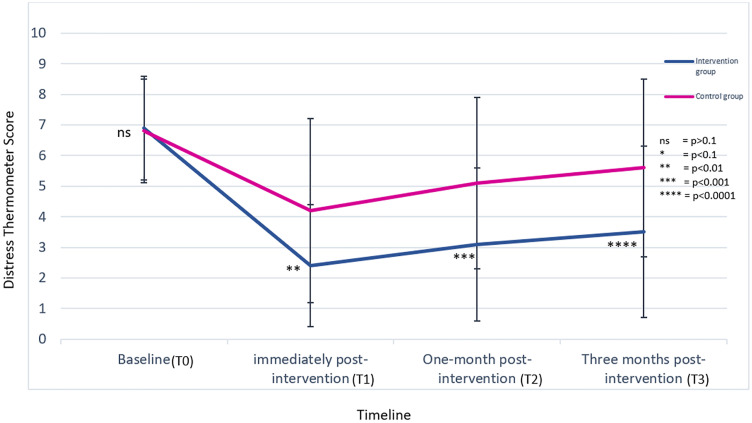
Comparison of the Distress Thermometer mean scores, between and within intervention and control groups. Note - Error bars represent the Standard deviation (SD).

The comparison of the Distress Thermometer scores within and between the intervention group and control group at T_0_, T_1_, T_2,_ and T_3_ is presented in Fig 2.

According to Fig 2, the Distress Thermometer mean scores showed no significant difference at the baseline between the intervention group and control group (p = 0.7). In all post-intervention assessments, the Distress Thermometer mean score was lower in the intervention group than the control group and was statistically significant (T_1_ = 2.4 [2.0] vs. 4.2 [3.0] p = 0.001, T_2_ = 3.1[2.5] vs. 5.1[2.8] p = 0.001, T_3_ = 3.5[2.8] vs. 5.6[2.9] p = 0.0001).

### Quality of life

**Fig 3:**
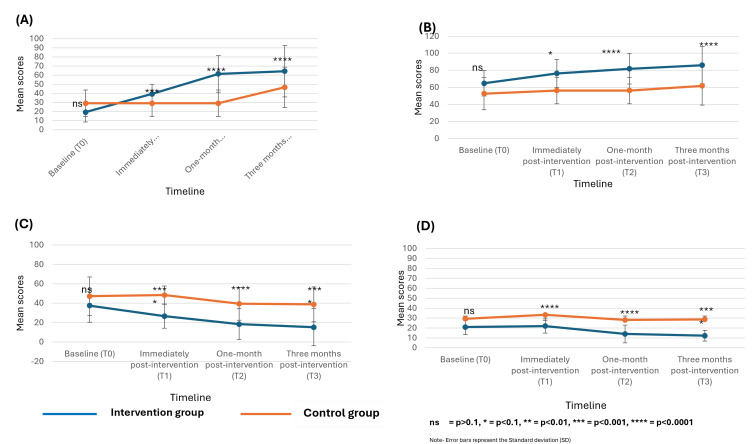
Comparison of the scores of (A) Global Health Status/ Quality of Life, (B) between Functional Status (C) Symptom Status, (D) H&N 35 Module between the intervention group and control group.

The comparison of the quality of life sub scale is presented in (Fig 3).

There was no significant difference in the baseline mean scores of all quality of life dimensions between the intervention group and control groups (p = 0.1). There was a higher mean score of all dimensions of quality of life in the intervention group compared to the control group at all post-intervention time points (GHST_1_ = 39.2[10.7] vs. 29[14.6], p = 0.001; GHST_2_ = 61.3[20.2] vs. 29[14.6], p = 0.0001; GHST_3_ = 64.2 [28.3] vs. 46.5[22.3], p = 0.0001; FST_1_ = 76.3[16.5] vs. 56.3[15.5], p = 0.1; FST_2_ = 81.8[17.9] vs. 56.2[15.5],p = 0.0001; FST_3_ = 86[21.9] vs. 61.8[22.6],p = 0.0001 SST_1_ = 26.7[12.4] vs. 48.4[9.4], p = 0.0001; SST_2_ = 18.4[15.9] vs. 39.5[16.9], p = 0.0001; SST_3_ = 15.2[19.1] vs. 38.8[17.9],p = 0.0001; H&N1 = 22.0 [7.2] vs. 33.2 [9.7], P = 0.0001; H&N2 = 14.0 [9.0] vs. 28.1 [10.8], P = 0.0001; H&N3 = 12.2 [10.4] vs. 28.7 [11.7], P = 0.0001).

### Multivariable analysis

After controlling for potential confounders and baseline scores, participants in the intervention group reported an improvement in psychological distress of 2.26 points (95% CI 1.07 - 3.35) compared to those in the control group (Table 3).

**Table 3:** Effectiveness of the intervention on level of psychological distress and quality of life after controlling for confounders.

Outcome	Adjusted Mean Difference (β)	95% CI	p-value
Psychological Distress	2.26	1.07–3.35	0.0001
EORTC - GHS	17.1	6.9–27.3	0.001
EORTC - FS	23.6	5.7–41.5	0.01
EORTC - SS	23.2	5.6–40.8	0.01
EORTC – H&N35	16.8	6.8–26.8	0.001

* Estimates are derived from separate linear regression models for each outcome. All models are adjusted for baseline outcome score and potential confounders, including, age, sex, ethnicity, level of education, status of employment and stage of the cancer. The β estimate represents the adjusted mean difference in outcome scores between participants in intervention group and those in the control group at T_3_ (final follow-up).

We have added this section - After adjusting for confounding variables, the sub-dimensions of quality of life demonstrated significant differences at T3 in the intervention group compared to the control group showed an improvement of: Functional Status 23.6 (95% CI = 5.7-41.5) (p = 0.01), Global Health Status 17.1 (95% CI = 6.9-27.3) (p = 0.001), Symptom Status 23.2 (95% CI = 5.6-40.8) (p = 0.01) and H&N35 16.8 (95% CI = 6.8-26.8) (p = 0.001).A post hoc analysis revealed no significant difference between the outcomes of the COVID -19 affected participants and unaffected participants (S5 Appendix). The mean delay in delivering the intervention sessions due to COVID-19 was 6 days.

## Discussion

This study suggests that the early palliative care intervention package may improve psychological distress and quality of life among patients with oral cancer immediately after, one month after, and three months after the intervention compared with standard cancer care. However, as separate analyses were conducted at each timepoint without modeling repeated measures, these findings should be interpreted with caution.

To the best of our knowledge, this is the first study on providing early palliative care for patients with oral cancer in Sri Lanka. The systematic review carried out in 2008 discovered 22 trials on specialized palliative care from 1984 to 2007 half of which were exclusively on cancer patients [37]. It suggested that these studies examining specialized palliative care have often been limited by methodological shortcomings such as control group contamination, high attrition, and inadequate comparison groups. In our study, we sought to enhance methodological rigor by implementing measures to minimize control group contamination and reduce participant attrition—for example, by conducting the intervention and control phases at different hospitals and by maintaining close follow-up with participants throughout the study period.

Although a randomized controlled trial would have provided the highest level of evidence, randomization was beyond the scope of this preliminary study. Therefore, a quasi-experimental design was selected as the most feasible and contextually appropriate approach for this first study of its kind in Sri Lanka. The findings from this work could inform the design and implementation of future randomized trials.

The formulation of the novel intervention package followed a thorough scientific process stipulated by the UK Medical Research Council for the development of complex interventions which assured the scientific rigor of the intervention package [30]. The intervention was a multi-component package that addressed six of the eight domains identified by the National Consensus Project on Clinical Practice Guidelines for Quality Palliative Care [38]. Especially, the intervention package utilized the existing resources in the health system by including Public Health Nursing Officers (PHNOs) who were already involved in the healthcare delivery. This favors the implementation and sustainability of the novel package in the low-resource health system in Sri Lanka. The timing of the intervention sessions—before surgery, and at one week and three weeks after hospital discharge—was selected to minimize heterogeneity among participants. Although discharge dates varied between patients, using standardized time points from the date of discharge helped maintain comparable conditions and reduce potential confounding bias. Each session averagely took 2 hours.

Our selection of quality of life as one of the outcomes of this study is supported since the ultimate goal of providing palliative care is to improve quality of life [15]. Selecting the psychological distress of the patients as the other main outcome of interest could be justified as there are proven adverse effects of untreated psychological distress on the cancer journey [5–7].

The key findings of this study indicate that participants in the intervention group experienced improvements in both psychological distress and quality of life across the study period, compared to the control group. Although this study does not establish a causal relationship between these two outcomes, the concurrent improvement observed aligns with previous evidence suggesting that lower psychological distress is often associated with enhanced quality of life among patients with cancer [39,40].

In our study, the intervention participants’ greater improvements in psychological distress and quality of life could be explained through the process indicators (S3 Appendix). The acceptance and implementation of early palliative care were higher in the intervention group as was evident through the results of process indicators: the percentage of patients who were aware of the information of the cancer journey patients who were following a diet plan, patients who received the financial allowance patients who had their pain managed patients who followed any type of mindful-based practice daily The slight improvements seen in the control group’s main outcomes could be also supported by the results of the process indicators since for each component of the novel palliative care intervention package there is some level of reception in the control group as well through the routine healthcare system in Sri Lanka where early palliative care is provided in an ad hoc manner.

Concerning the quality of life dimensions, the results demonstrated a significant improvement in the symptom status in the intervention group. This is an uncommon finding since a systematic review of palliative care effectiveness analyzed 14 studies that measured symptom intensity using a variety of scales, only 1 showed an improvement [37]. It is possible that the current study finding could be due to the participants being just after the diagnosis and the initial cancer treatment itself contributed to the improvements of the symptom status. However, the current study findings can be supported by a randomized control trial conducted on patients with advanced cancers providing early palliative care showed that at 4 months post-intervention, there were significant differences in change scores of quality of life, symptom severity, and satisfaction with care. This study has more power in the findings being a randomized control trial. Although the target group was different in the two studies, since this study focused on patients with advanced cancers of lung, gastrointestinal, genitourinary, breast, and gynaecological the current study was only on patients with oral cancer, the findings are in line [17].

There are several studies which have failed to show an impact on the quality of life by palliative care interventions. This may be due to a lack of comprehensiveness in the study design, and the presence of only one or two of the domains of the quality of life in the intervention [41–43]. A study carried out on patients with head and neck cancer in India did not show significant improvements in the quality of life after 1 month, 2 months, and three months of randomization [21]. The author attributes this result to the fact that the control group also received a comparable level of palliative care through the routine healthcare system. Also, another reason for the different findings could be that the participants of this study had been patients with stage IV disease or recurrence not amenable to curative treatment and planned for palliative intent chemotherapy whereas the current study included patients presenting at all stages of the disease.

### Clinical implications

The findings of this study suggest that the early palliative care intervention package developed and tested here has the potential to be integrated within the existing health system to support patients with oral cancer. In particular, Public Health Nursing Officers could play a key role in linking tertiary care hospitals with community-level services to address patients’ psychological distress and improve their quality of life. However, before large-scale implementation, these findings should be confirmed through further studies, ideally using randomized controlled designs and larger, more diverse samples.

### Strengths and limitations of the study

Our study has several strengths to be acknowledged. The response rate being 100% and there was no loss to follow-up. This is an outstanding finding of this study and could be attributed to several factors. The participants are just after the diagnosis. Therefore, the enthusiasm for follow-up care is higher than the patients who are in the latter part of the cancer journey. Also this was likely influenced by the absence of structured supportive care services in the Sri Lankan health system, which made the intervention particularly valuable and welcomed by patients. Stringent measures, such as aligning appointments with clinic visits and persistent phone reminders, would have also minimized dropouts. In the Sri Lankan context, where healthcare is provided free of charge through a publicly funded system, patients tend to show strong trust and compliance toward hospital-based care and research activities. The hierarchical doctor–patient relationship and relatively low levels of patient empowerment may also contribute to this tendency. These contextual factors, inherent to the local health system, may partly explain the high level of participant compliance observed in this study.

We acknowledge a few limitations in the study. The selection of a non-randomized controlled trial has introduced its own biases, however being the first study on early palliative care provision for patients with oral cancer in Sri Lanka this could be considered as a rational starting point for further research in this arena. The study could be under-powered for the psychological distress outcome because the calculation was not specifically tailored to it. We used the presence of an accessible Public Health Nursing Officer to allocate the participants to the intervention and control groups.. In Sri Lanka, Public Health Nursing Officers are attached to the Healthy Lifestyle Clinics in Base Hospitals and provide services to the communities within those hospital catchment areas. Consequently, only patients residing in areas covered by a Base Hospital received the intervention, while those living farther away did not have access to these services. Because the presence of Base Hospitals is often associated with areas of relatively higher socioeconomic development, this uneven distribution of PHNOs may have introduced a socioeconomic status–related bias in the delivery of the intervention.

Although data distributions were skewed, results were presented as means (SD) to allow comparability with previous studies using the same outcome measures. Nonparametric confidence intervals were not estimated, which may limit the precision of reported effect sizes. Also the analyses of the outcomes were based on separate between-group comparisons at each timepoint and a baseline-adjusted regression at the final follow-up. This approach does not fully account for the correlation of repeated measures or adjust for multiple testing, which may increase the risk of Type I error. More advanced longitudinal modeling techniques, such as mixed-effects or difference-in-differences analyses, could have provided a more comprehensive understanding of the intervention effect over time. Therefore, findings should be interpreted with caution.

The measures used to assess the outcome indicators are both validated to Sri Lanka. However, the process indicators developed in the current study only considered content and face validation. Therefore, this could be a limitation and it is recommended to follow a comprehensive validation for the process indicators in future studies.

We identify the exclusion of any patient who was unable to communicate in Sinhala as a limitation since Sri Lanka is a country with multi-ethnicity the ethnic and racial representation would not have been present. Hence, we recommend the necessity of replicating this study with more diverse populations.

## Conclusions

The early palliative care intervention package showed promising effectiveness in improving psychological distress and quality of life among patients with oral cancer in Sri Lanka. Public Health Nursing Officers may play a valuable role in linking tertiary care hospitals with community-level services to support patients’ overall well-being. However, these findings are based on a relatively small sample from the Sri Lankan population and may not be generalizable to other settings. Future studies with larger and more diverse samples across different geographical and cultural contexts are recommended to further evaluate the intervention’s effectiveness.

### Patient and public involvement

During the development of this intervention the inputs of the patients were taken into consideration by conducting in-depth interviews with the patients. The findings of the study will be disseminated through the patients with oral cancer who come to the tertiary cancer care units in Sri Lanka.

## Supporting information

S1 Appendix TREND statement checklist. (DOCX)

S2 Appendix Early palliative care intervention package for patients with oral cancer in Sri Lanka. (PDF)

S3 Appendix The TIDieR checklist. (DOCX)

S4 Appendix Process indicators comparing the reception* of early palliative care between the intervention and control groups. (DOCX)

S5 Appendix Impact of COVID-19 to the study. (DOCX)
