# Supplementary material for: Early palliative care for patients with oral cancer in Sri Lanka: A non-randomized controlled trial
Source: PLOS Glob Public Health. 2026 Mar 9;6(3):e0005985. doi: 10.1371/journal.pgph.0005985 (PMC12970869; doi:10.1371/journal.pgph.0005985)
Supplement: S2 Appendix — (PDF) [file pgph.0005985.s002.pdf]

Appendix II

Early Palliative Care Intervention Package for Patients with Oral Cancer in Sri Lanka

Content

A. Objective of the intervention package
B. Components of the intervention package
C. Setting of the intervention package
D. Recipients of the intervention
E. Deliverers of the intervention
F. The timing of the sessions of the intervention package
G. Details of the intervention
H. Material used for the intervention package
I. Monitoring of the intervention

A. Objective of the intervention package

To improve the psychological distress and quality of life of patients with oral cancer who are waiting for surgery.

B. Components of the intervention package

1) providing information
2) addressing acute and functional issues
3) nutritional care
4) psychological support
5) mindfulness therapy
6) coordination of the financial allowance

1) **Providing information**

- The modes of providing information were: through face-to-face discussion with the PI and Public Health Nursing Officers over three sessions of the intervention delivery, a simple booklet in clear language (Annexure a), and a short informative and motivational video clip shown to the patient and the caregiver.
- The development of the booklet and the video clip followed a scientific process [26] and were pretested. Special attention was paid to low levels of education and consequently low levels of health literacy of patients with oral cancer in the Sri Lankan context.
- The information provided included;
    - the status of the condition
    - the surgery
    - life after surgery - information on nutrition, wound care, oral care, mental health, rehabilitation and empowering the patient to face society and become an ambassador of goodwill/role model.
- Prior to providing information, the PI probed into the extent of the patient's current knowledge about his/ her condition. The PI thoroughly went through the patient's available records and had a discussion with the ward House Officer before providing any extra information to the patient. At the end of the video clip, the patient was given time to ask any queries and clear the doubts. Finally, the patient was provided with a booklet containing the information.

2) **Addressing acute and functional issues**

- The focus was on pain management, wound care, and rehabilitation of the basic functions.
- Any lapses in controlling pain or wound care were identified. The required corrective advice was given to the patients. If further management was needed referrals were done.

- Special emphasis was given to pain management since it directly affected the functional issues. The patient was given a pain scale to be maintained since the discharge from the hospital after surgery. This scale was reviewed each time the PI and Public Health Nursing Officer met the patient. The issues identified regarding pain were initially addressed by educating the patient and offering remedies to lessen the disability. Furthermore, an explanation of the treatments available was provided. If needed patient was referred to health professionals for further management. The relevant officers to whom the patient was referred were personally informed regarding the referral to minimize unwanted hazel.

3) **Nutritional care**
   - At the 1st session the patients were provided with information on the importance of maintaining proper nutrition after surgery.
   - A diet plan was provided to the patient in consultation with the nutritionist. A special request was made to the nutritionist to always stick to a low-budget dietary plan that fits the patient’s financial status. The patient was followed up for compliance with the diet plan and the needed diet counseling was carried out during the following two sessions conducted at his/her residence.
4) **Coordination of the financial allowance**
   - The needed coordination to a financial allowance which was already in place from ‘Grama Sewaka Niladari’ was accomplished and it was ensured that the patient received it at the right time without hazel.
   - Furthermore, the patient was introduced to alternative self-employment methods like sewing, and weaving if he/she did not have any income source.
5) **Psychological support**
   - Aimed at the patient and the family members,
     - to overcome the distress caused by the disease itself
     - to manage social and relationship issues
   - Psychological support included
     - Ventilation of emotions

- Information and education
- Problem-solving counseling
- Maintaining hope

6) Mindfulness therapy

- The steps involved,
    - Explanation of the relationship between the mind and the body eg. recalling what happens to the body when one gets angry.
    - Explain the advantages of being fully present in the current moment, especially for a patient with cancer.
    - Help the patient to be present in the current moment by focusing on anything that the patient is currently involved with.
    - Carry out one simple (5 mins) session focusing on breathing with the patient, to begin with, and share the experience and difficulties faced while doing the session.
    - Accept that it would be difficult at first, but the 'mind' could be trained as it has massive unharnessed potential.
    - Gradually help the patient to train the mind to focus on daily activities, breathing, and other mindfulness practices, feelings and finally to the method of 'letting go'.

C. Setting of the intervention package

The intervention needed to link the hospital and community. The first session was conducted at the tertiary care unit in the ward and the subsequent two sessions were delivered at the community level (at the home of the patient).

D. Recipients of the intervention

The following inclusion criteria were made based on the findings of the literature review, and case-control study, to control the confounders.

Inclusion criteria were knowledge of the definitive diagnosis communicated to the patient, presence of psychological distress (receiving a score of 4 or above after screening with the

Sinhala version of the Distress Thermometer), awaiting surgery as the first treatment modality, married with children and had at least one caregiver who was a family member and the ability to communicate and read in Sinhalese. The recurrent oral cancers, psychiatric diagnosis of an illness, and those patients who received any early palliative care intervention were excluded from the study.

The caregivers were involved in all the sessions.

E. Deliverers of the intervention

- **The PI**
    - Primarily a dental doctor, followed by postgraduate studies in Public Health delivered the 1st session of the intervention package at the hospital where the patient received cancer care. She underwent few capacity-building programmes in habit intervention, counseling skills, pain management, clinical psychology, and Mindfulness Therapy.
    - Continuous monitoring and training by a consultant psychiatrist was provided.
- **The Public Health Nursing Officer**
    - Primarily a nursing officer who has experience of a minimum of 6 years and volunteered for the post of Public Health Nursing Officer. A 6-month course on palliative care, elderly care, and care for non-communicable Diseases is given.
    - A special training in two sessions was given to deliver the early palliative care intervention package. This was followed by another virtual training session just before the delivery of the intervention package (Annexure b)
    - A hand book was given to all Public Health Nursing Officers where it gave specific guidance to deliver the package. (Annexure c)

F. The timing of the sessions of the intervention package

- The initial session was carried out before the surgery at the clinic when the patient got admitted to the ward for the surgery. The second session was carried out after 1 week of discharge from the hospital. The third session was carried out following a two-week gap to the second session.

G. Details of the intervention

- The intervention was delivered face-to-face individually. The summary of the novel intervention package is presented in the following table a.
- The specific delivery instructions were provided by the handbook which was given to the Public Health Nursing Officers.
- Delivery of the intervention needed to be in line with the instructions given in the booklet but, the PHNO was given instructions to make minor changes based on the basic framework of the intervention, to serve the need of the patient.

Session no.	Time of the intervention	Setting of the intervention	Providers of the intervention	Content of the session
1	After diagnosis before the surgery	Tertiary care Hospital (ward)	Dental Doctor (PI)	- Providing Information<br>- Nutritional care<br>- Psychological support<br>- Mindfulness therapy<br>- Coordinating the financial allowance
2	1 week after being discharged following surgery	Residence of the patient	Public Health Nursing Officer	- Address acute and functional issues<br>- Nutritional care<br>- Mindfulness therapy<br>- Psychological support<br>- Coordinating the financial allowance
3	3 weeks after being discharged	Residence of the patient	Public Health Nursing Officer	- Address acute and functional issues<br>- Nutritional care<br>- Mindfulness therapy<br>- Psychological support<br>- Coordinating the financial allowance

Table a- Early palliative care intervention package for patients with oral cancer

H. Material used for the intervention package

- A training module to increase the capacity of the PI and Public Health Nursing Officers was developed with the inputs of experts and scientific evidence.
- A handbook was given to the Public Health Nursing Officers giving specific advice on how to deliver the intervention package
- A booklet providing information about the cancer journey to the patient. This was given to the patient during the first session of the early palliative care intervention package.
- A video clip providing information about the cancer journey to the patient. After providing information verbally and by handing over the booklet a video clip was shown to the patient individually. This was done through a laptop. The patient was given the opportunity to ask questions after providing information in these three modes – verbally, through the booklet, and through the video clip.

I. Monitoring of the intervention

- The intervention package that was delivered was monitored throughout by the PI. This was carried out by two methods namely continuous supervision of the Public Health Nursing Officers through contacting them over the phone and second by contacting the patients throughout the intervention.
- Each Public Health Nursing Officer was initially contacted to hand over the patient to deliver the intervention. Then throughout the intervention which is before and after each session, the Public Health Nursing Officer was contacted and an assessment of the session delivered to the patient was done. Once the Public Health Nursing Officer finished delivering the intervention, the patient was contacted by the PI. All together the patients were contacted thrice through the telephone i.e., just after the intervention, 2 weeks and 2 months after.
- The conversations with Public Health Nursing Officers provided needed corrective measures and ways to overcome limitations. During the conversations with the patients, their compliance with the given instructions was checked and the needed follow-up assessments during clinic visits were arranged.
- Besides the above standard contact points, Public Health Nursing Officers were informed to contact the PI when the need arose to deliver the intervention package. A follow-up sheet

for each patient was given by the PI at the first session of the intervention. This sheet was filled at each contact with the patient by the Public Health Nursing Officers. With the data from this sheet, a process evaluation framework was developed which helped to monitor and evaluate the effectiveness of the package.

Annexure a

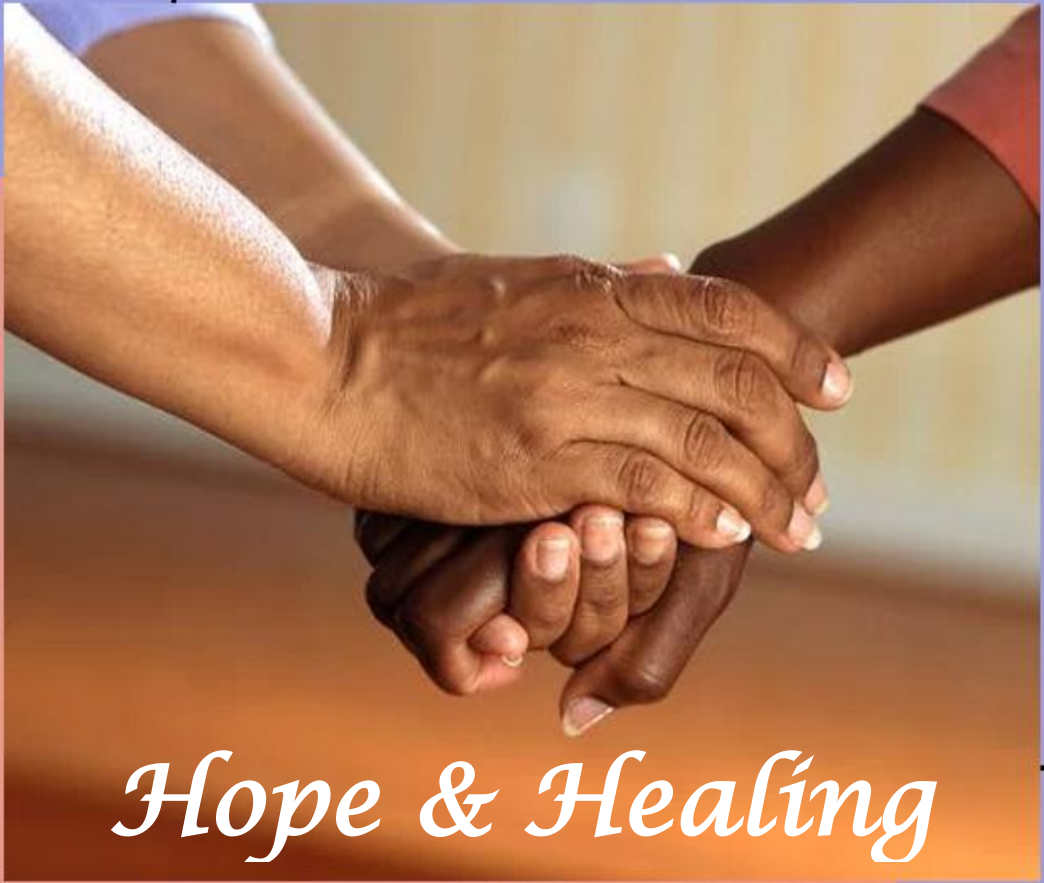

Hope & Healing

A guide to win your mouth cancer journey

We know that finding out that you have cancer makes you feel afraid, anxious and worried. We understand that it brings many changes for you and your loved ones. We are with you.

Take a little time to read this book carefully. It will help you to,

- answer many questions that are bothering you now
- understand what you will experience in your future
- understand who is out there to help you through this journey

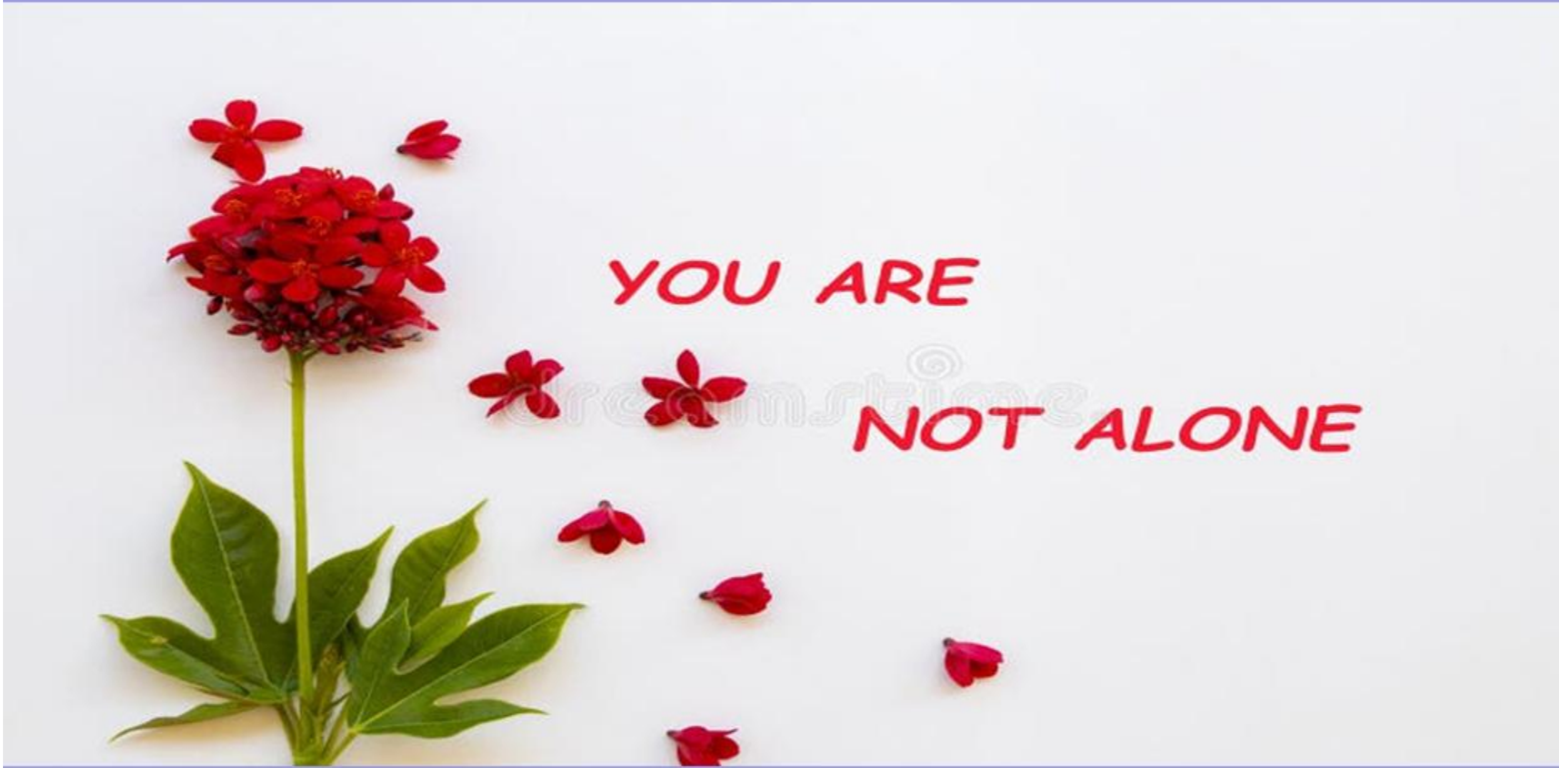

There are many people to help you go through this journey…

Contents

Page no

5 Just Diagnosed

18 The challenges ahead

57 For your loved ones who take care of you

61 You can help others

Just diagnosed

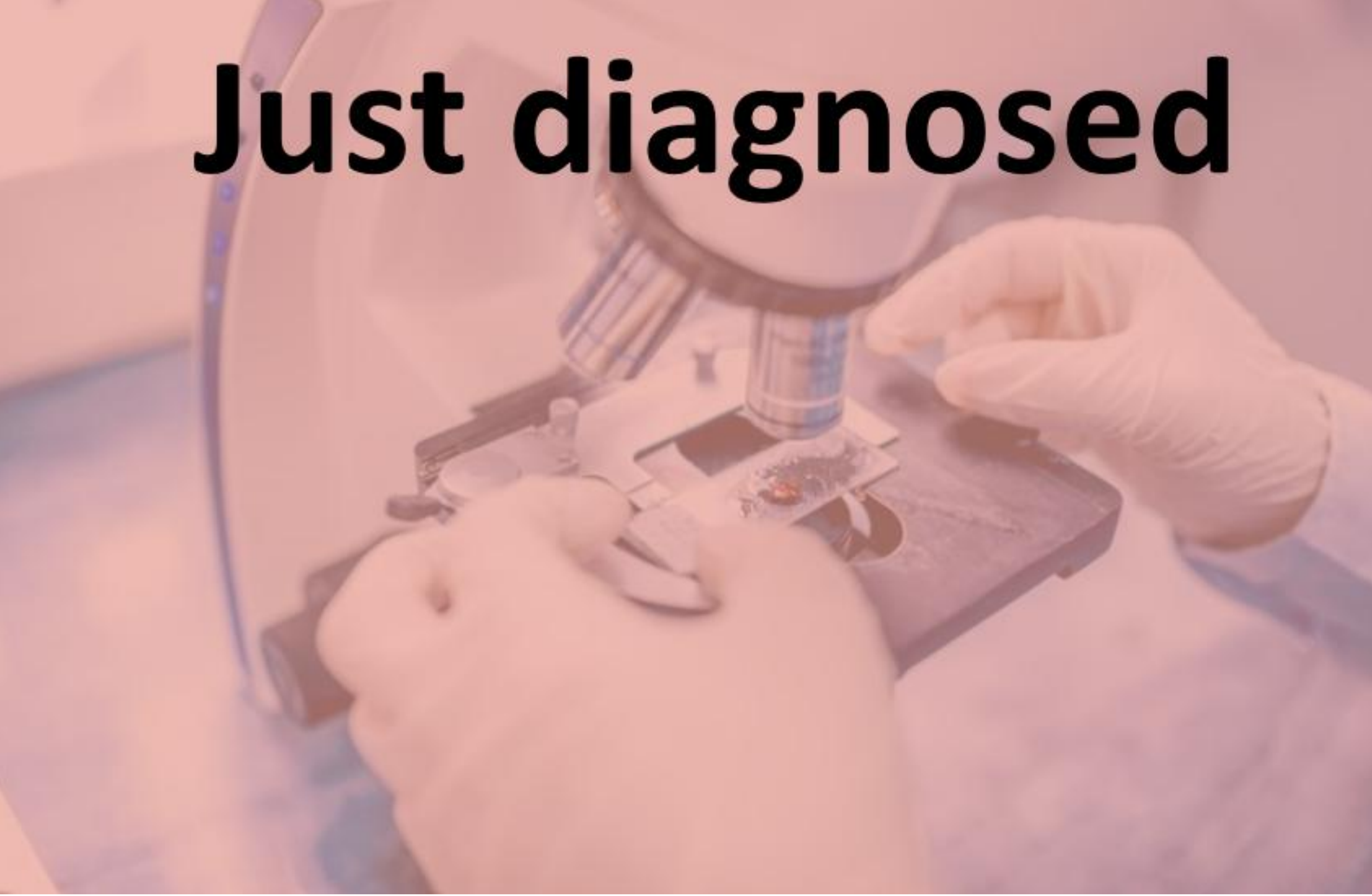

The Oral Cancer Journey

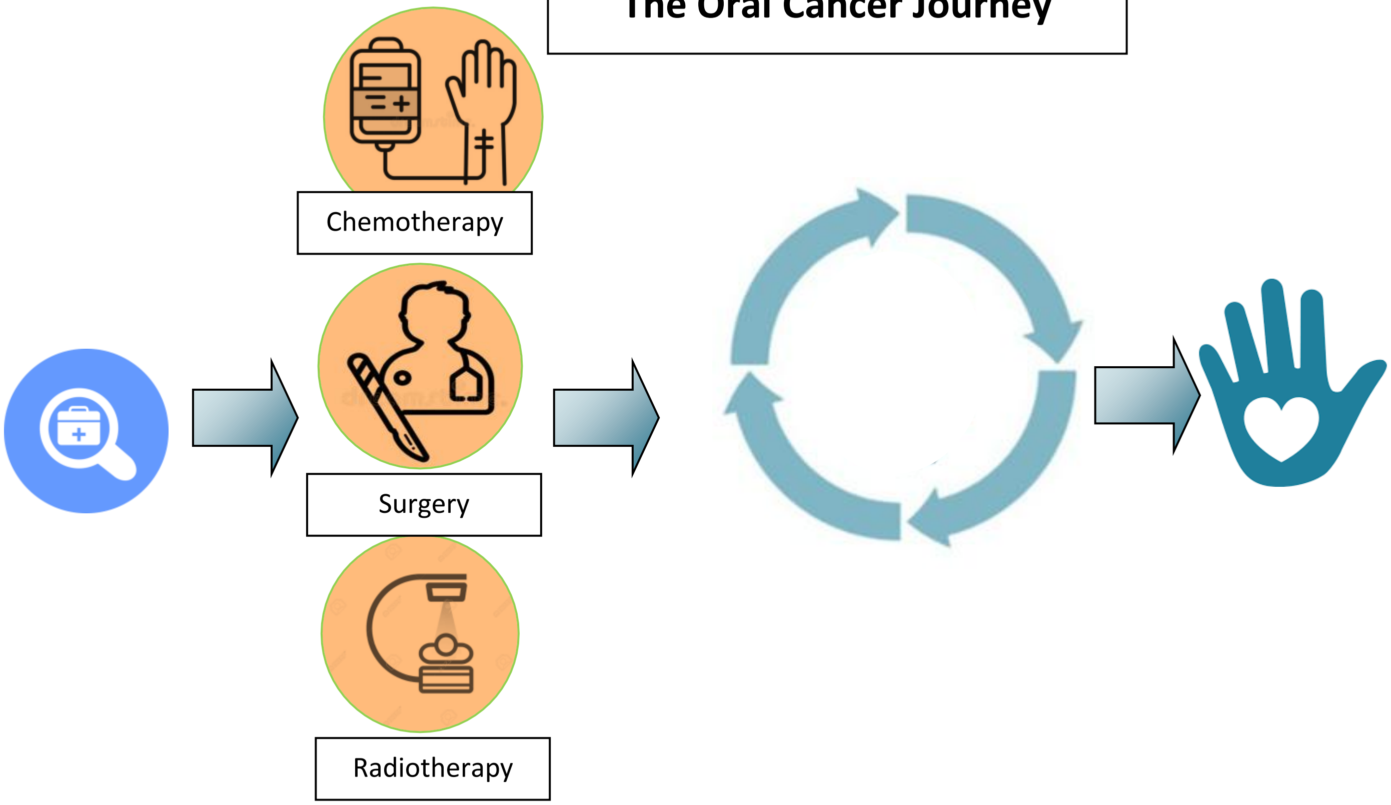

A- Diagnosis

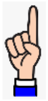

You are here

B- Different types of treatment available

C- Treatment cycles

D- Living well after treatment is over (Survivorship)

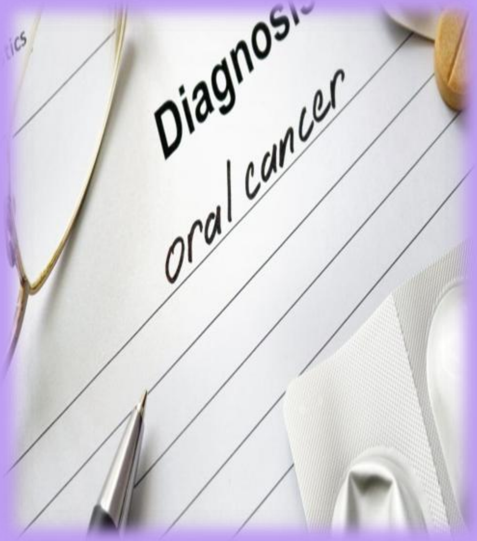

A- Diagnosis

When you are first told that you have mouth cancer you may have lot of questions which is normal.

Knowing what will happen to you properly will help you to settle down.

This section will tell you about your cancer journey starting from the diagnosis

A- Diagnosis

Now you know about your condition. You can ask for more details from your hospital team because it will help you to know where you stand which will relax yourself and your loved ones.

What is mouth cancer?

Mouth cancer is an unusual set of cells in the mouth that grows without control and crowds the normal cells. This makes hard for the body to work in its normal manner.

Stage of your cancer?

You need to know the stage of your cancer. It tells you how much your cancer has grown and the extent it has spread. You can ask your doctor to explain about the stage of your mouth cancer.

Why treating cancer is important?

If we do not treat the mouth cancer it can spread to your other parts of the body and cause problems. Also, treatments make your life easy. Removes pain and helps you to have a normal life.

Knowing exactly about your cancer will ease your stress and help you to cope peacefully. After knowing your diagnosis how will you talk about this to your loved ones? Does this bother you?

We understand that it could be. Here are some tips.

- Talk to your family about it when you feel ready. Take your time.
- Explain to them the type of cancer and the treatment plan you have discussed with your doctor.
- Share with the people whom you are most comfortable with. You do not have to tell everyone around you.
- Allow them to share how they feel and let them help you through the journey.

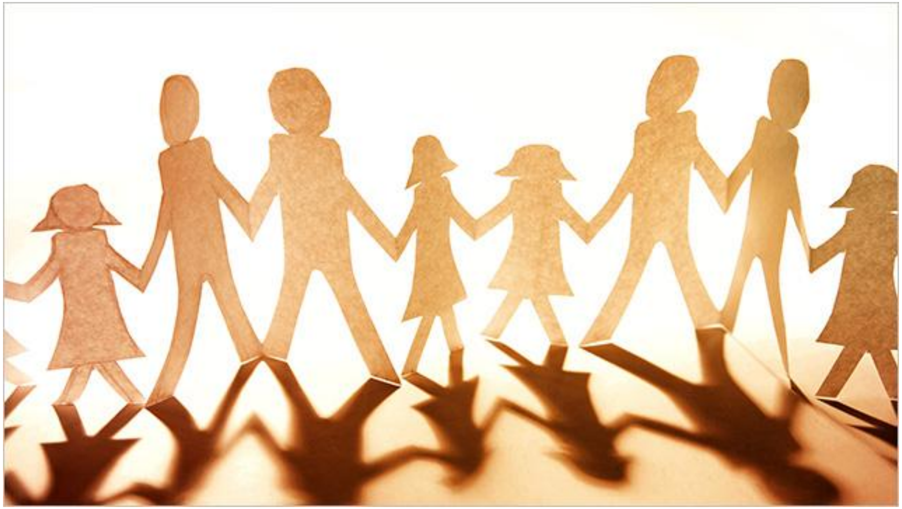

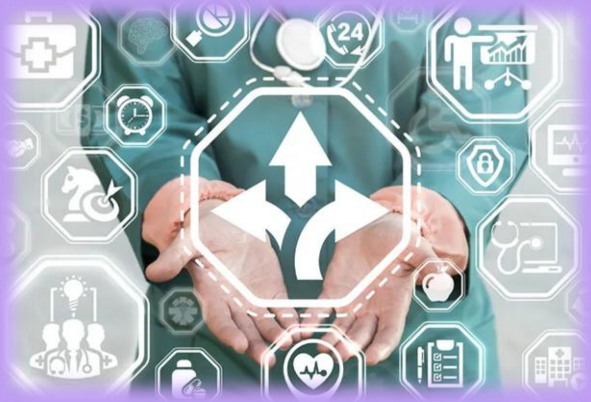

B- Different types of treatment available

You are now about to start your treatment journey.

This section will tell you about the common treatment types available to treat cancer.

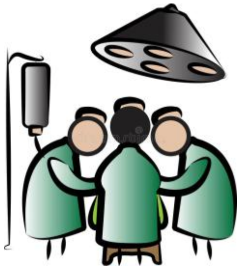

1. <u>Surgical Treatment</u>

Mostly this is the basic treatment option for mouth cancers. This involves removing the cancer parts. Your doctors plan carefully to ensure that they remove the minimal and will try to remake the defective regions, due to surgery to their best ability

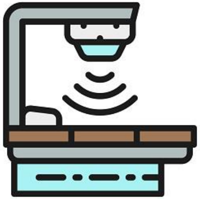

1. <u>Radiation Therapy</u>

The cancer cells are destructed using high-energy radiation. This treatment is known as ‘current treatment`, `light treatment` in the society. There is nothing to fear. This does not cause any pain or burning sensation. However, you might feel a slight discomfort.

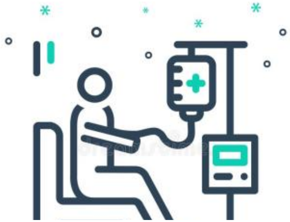

3. <u>Anti-Cancer Drug Therapy</u>

In some oral cancer cases this treatment method has been used. These drugs are administered orally (by mouth) or injected. It destroys cancer cells.

In all the above treatment methods, your doctors are always careful to minimize the damage caused to normal parts of your body. However, you will face a few difficulties for a while after treatments such as dry mouth, loss of appetite, difficulty in swallowing.

Remember, these difficulties are short term. They will disappear with time, and you will learn how to overcome them.

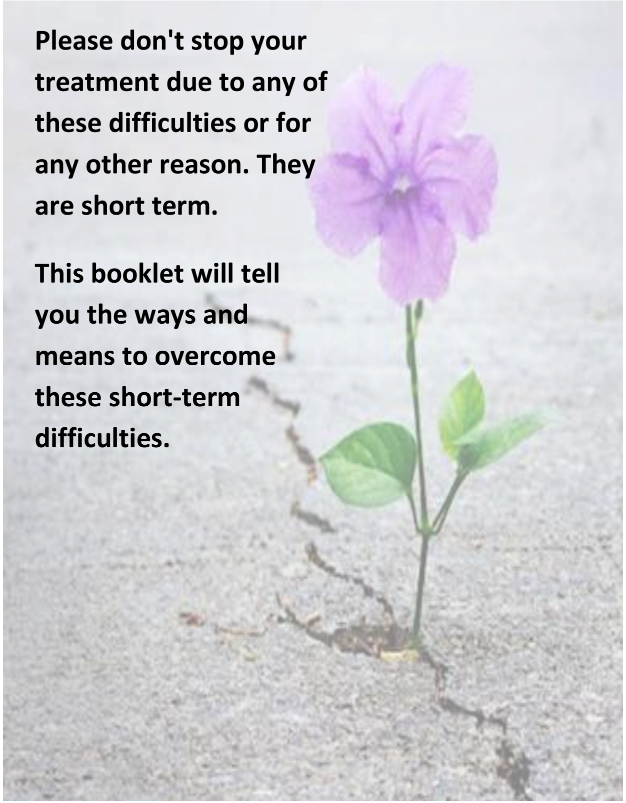

Please don't stop your treatment due to any of these difficulties or for any other reason. They are short term.

This booklet will tell you the ways and means to overcome these short-term difficulties.

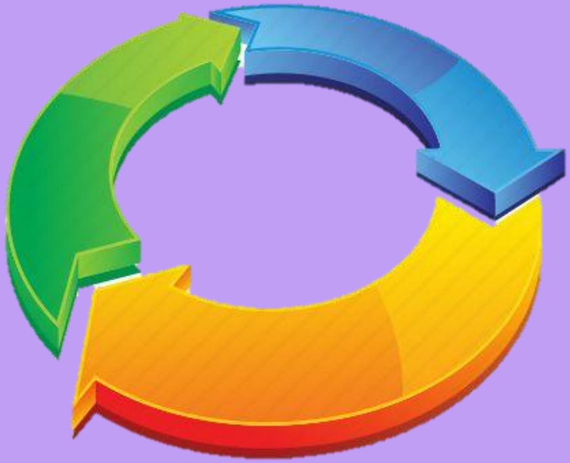

C- Treatment cycles

You have now finished the first cycle of your treatment journey. Now what?

Based on the condition of the illness and response to the treatments already given, your doctors will decide whether you need to go through the treatment cycle again.

Know that if your doctor recommends you go through a few more treatment cycles it is for your own good and by giving your maximum support to this decision the illness will subside faster.

Go through this journey patiently. All the temporary challenges you face will move away fast. Till then walk courageously using all the support given to you by your hospital staff, relations and friends.

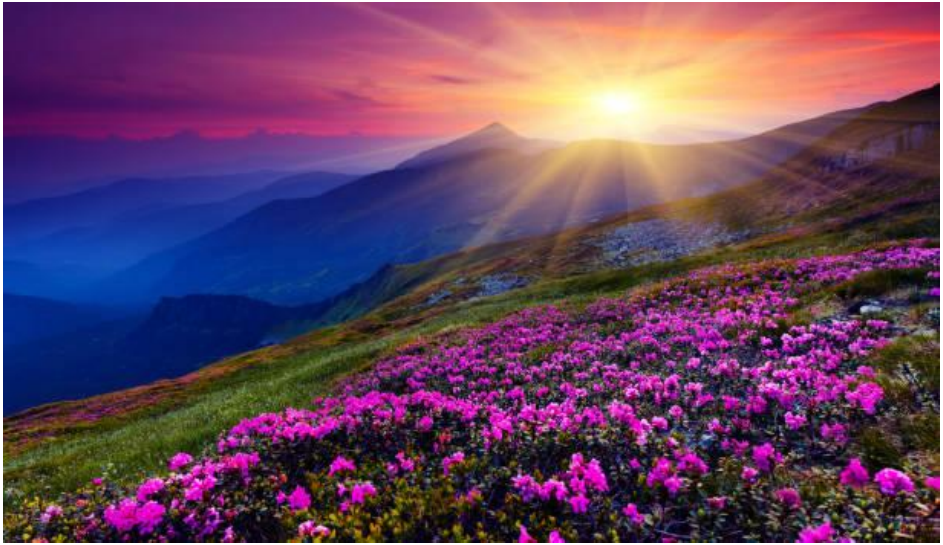

G

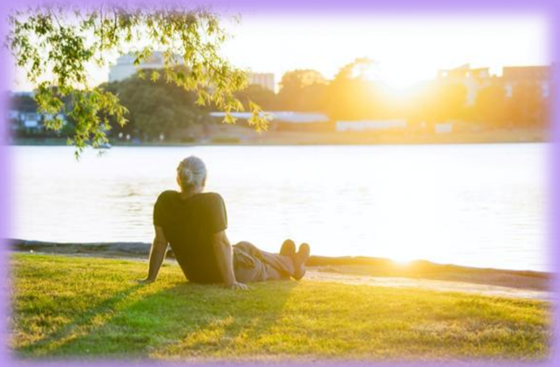

D- Living well after treatment is over (Survivorship)

You have now finished all the cycles of your treatment journey. What do you do now?

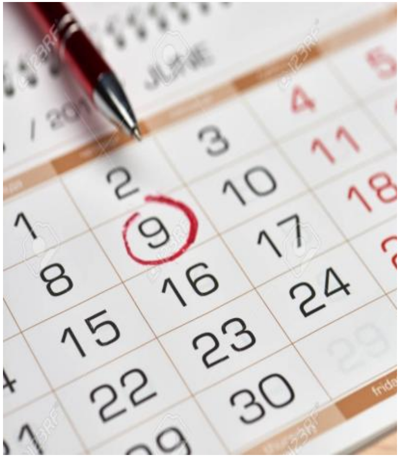

Do you need to go for follow-up clinic visits?

- This period is important! You are at a stage where your cancer has been controlled. It is very important to keep an eye on your cancer as well as your general well-being.

- Your health staff will give you the necessary clinic appointments to check you. Make this a priority and visit your clinic on the appointment date. During this follow-up period give your best compliance to all the instructions your health staff give you.

Will you get cancer again?

- Ask your doctor about the risk of getting cancer again from your doctor and what changes you should look out for. There is nothing to fear about getting cancer again if you find it early. Therefore, get to know about what signs and symptoms you will get and if you find any of these changes visit your clinic.

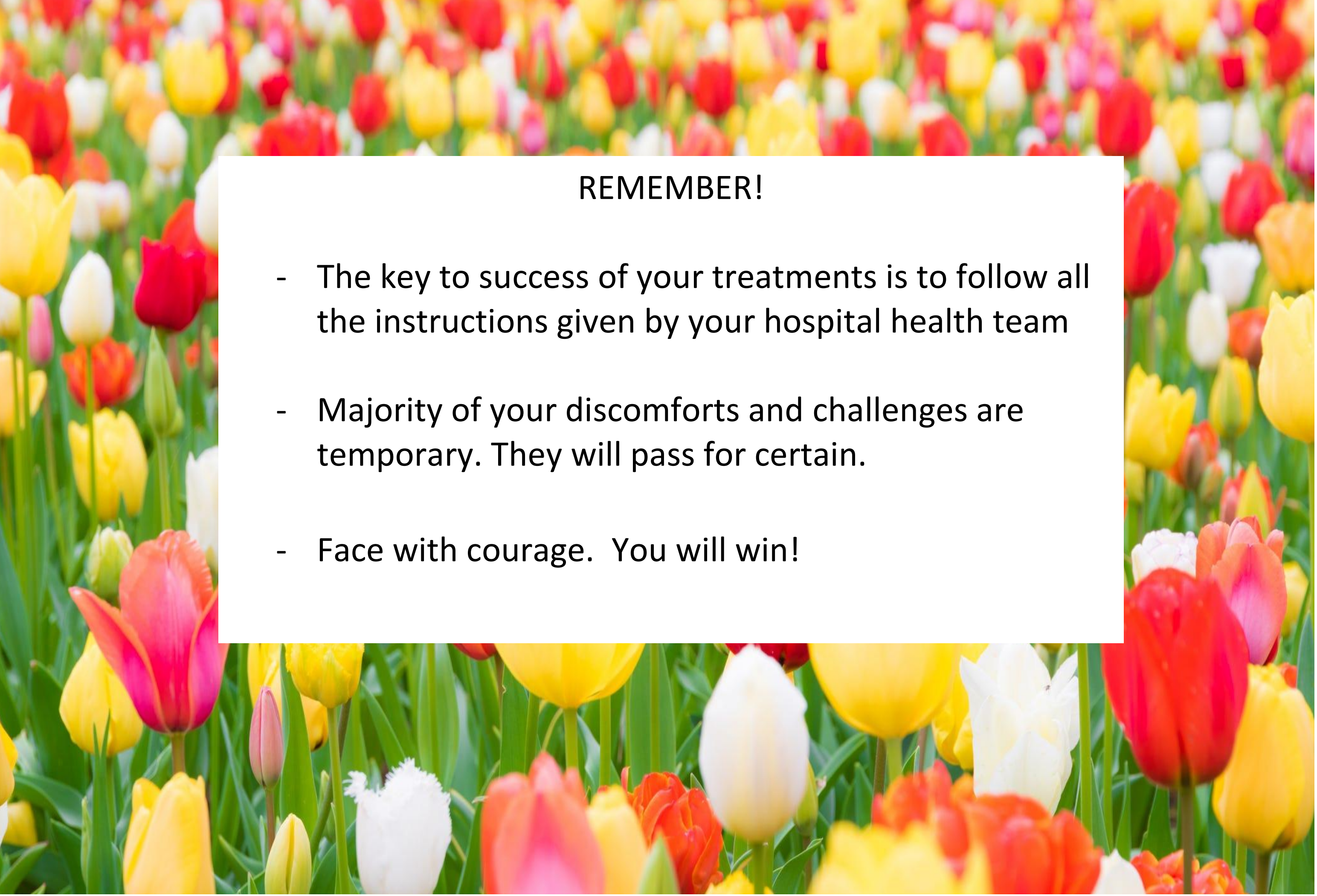

REMEMBER!

- The key to success of your treatments is to follow all the instructions given by your hospital health team

- Majority of your discomforts and challenges are temporary. They will pass for certain.

- Face with courage. You will win!

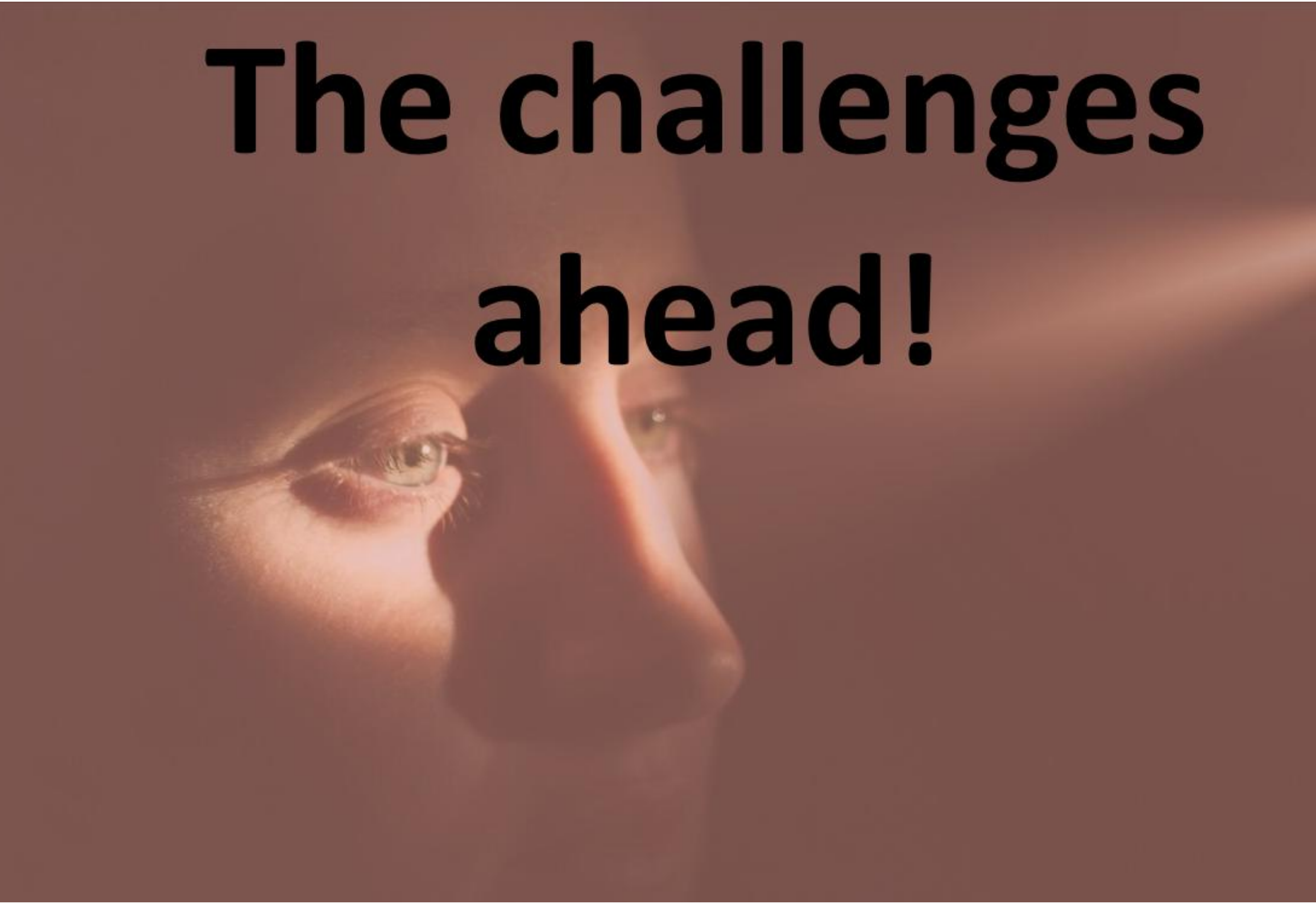
The challenges
ahead!

Now you know what your cancer journey would be. Let's go on to talk about the challenges you will face and tips to face them successfully.

You are a collection of MIND, SPIRIT/SOUL AND BODY. Cancer can only harm your body - that is temporarily too. It cannot damage your mind and spirit. Despite some temporary drawbacks, you can always be a winner! Therefore, don't let cancer to grab the 'whole you'!

Let's strengthen your mind and soul, during this time when your body prepares for a challenge. This will help your body to pass through this challenge easily and successfully.

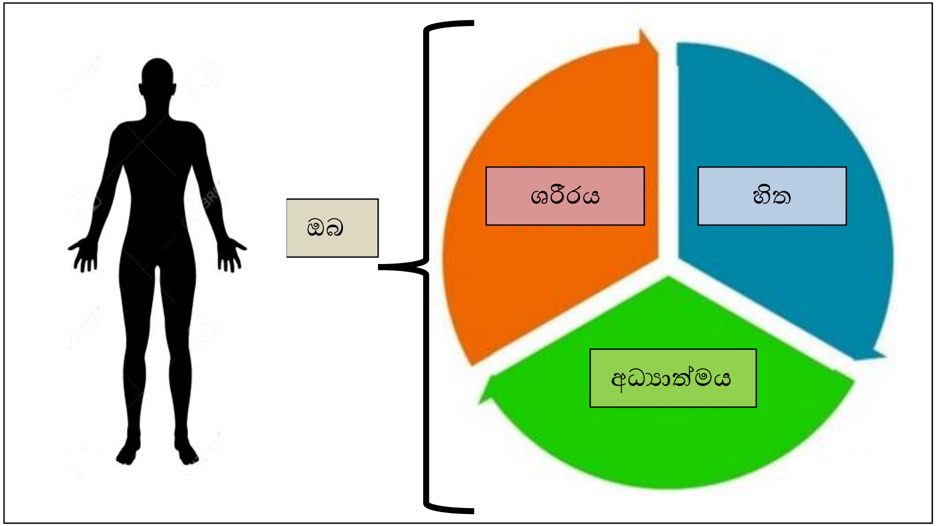

We can divide the challenges you will face into the following

1. **Physical challenges**
2. **Mental challenges**
3. **Spiritual challenges**
4. **Financial challenges**
5. **Social challenges**

Let's discuss each of these aspects in detail. The following chapter will give you the different challenges you will face and more importantly how to overcome them.

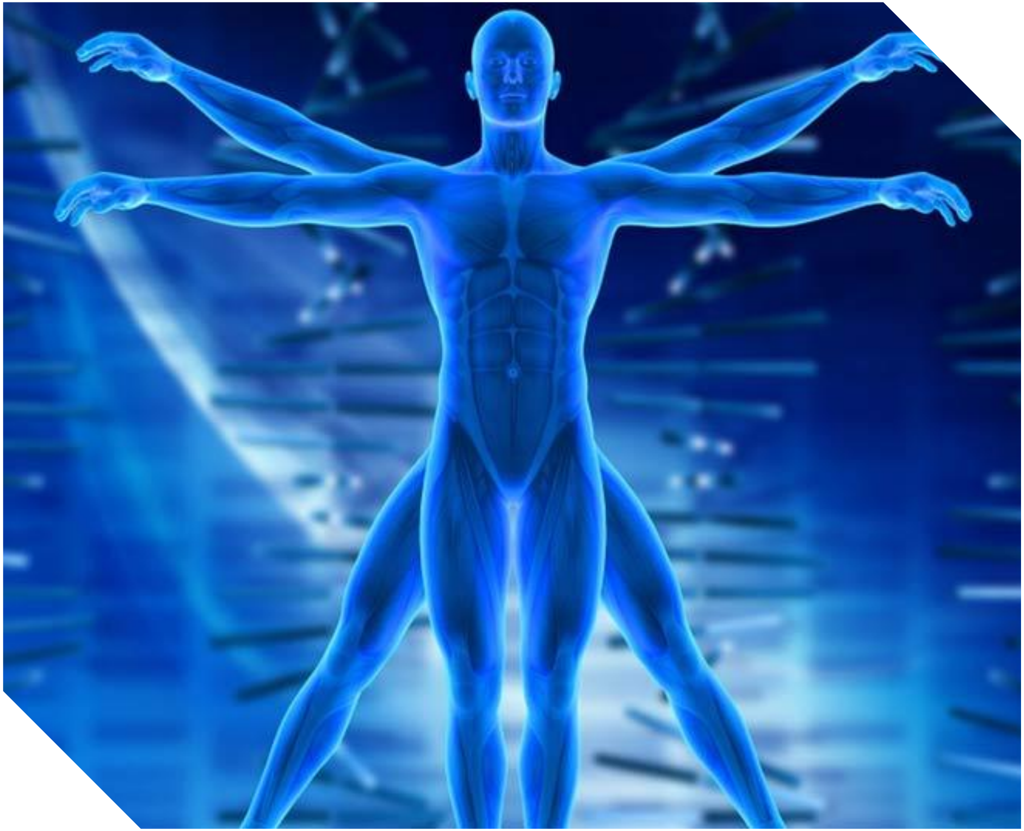

Do’s

- Take the prescribed medicine at the correct time, in the correct dose. This will address many physical discomforts.
- If discomfort is still present even after the medication, seek medical advice.
- Engage in your routine activities as much as possible. (As per the medical advice).

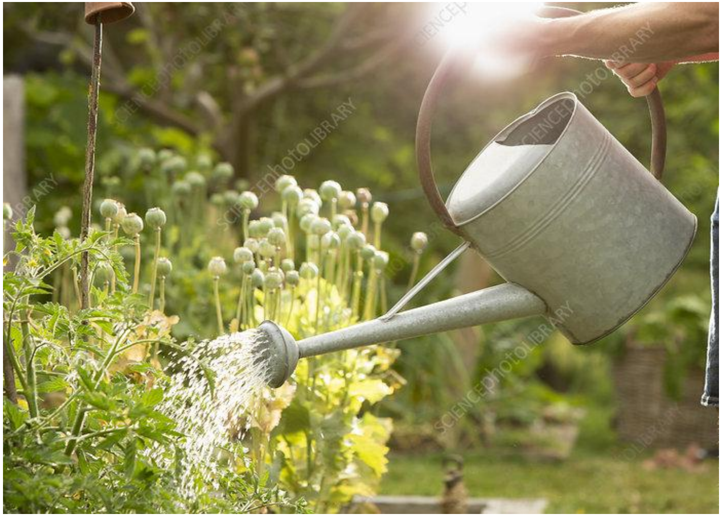

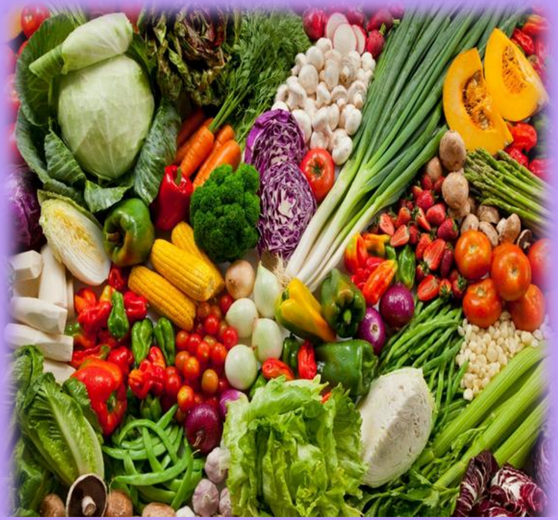

Proper nutrition

Your wish is to get better soon. The medication alone can't do the job. Getting the right nutrition is very important. Especially after the surgery. Try following these tips for a proper nutrition.

- The hospital might provide you with a diet plan. Please follow it at your best. If it is not possible to follow, due to your monetary issues, talk with your doctor and he/she will try to come up with a reasonable plan.
- Eat small amounts per meal and have several meals per day. (Two hourly)

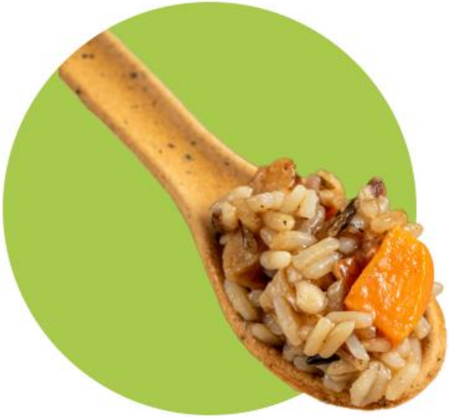

- You may find it difficult to eat solid food initially, but solid food is very important to get the proper nutrition. Therefore, firstly try to eat solid food try the following methods.

- Add Oil, Butter/Margarine while prepairing food.
- Blended & mashed food (Sweet Potatoes, Potatoes, Peas, Cowpea, Green gram, Corn etc.) will make your eating less uncomfortable.
- Take small amount into mouth at a time.
- Drink liquids (especially water) after eating solid food.

- If eating solid food is very much difficult, try semi solid food (like Kanji, yoghurt, curd, jelly, waṭalappan etc.)
- If taking semi solid food is also difficult, then you could go for liquids (like Milk, Barley, King coconut etc.).
- Even though taking liquid and semi-solid food is easy, try to eat solid food as much as you can.

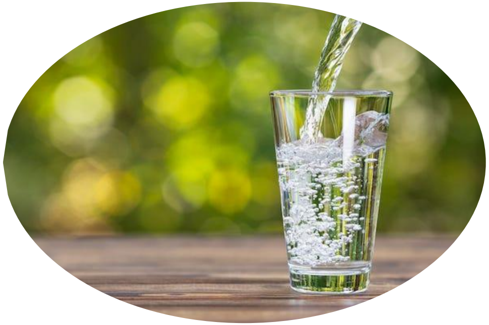

- Drink at least 8 glasses of water per day.
- Eat enough fruits & vegetables.
- It is normal to feel dryness in the mouth, burning sensation, lack of taste for some time. Therefore, minimize eating spicy, extremely hot or cold food.

- Prepare your food in attractive ways.
- You will recover the taste feeling after little time till then, place various flavors on the tongue & try to identify the tastes.
- Seek medical attention if you continue to have loss of appetite, nausea, or vomiting.

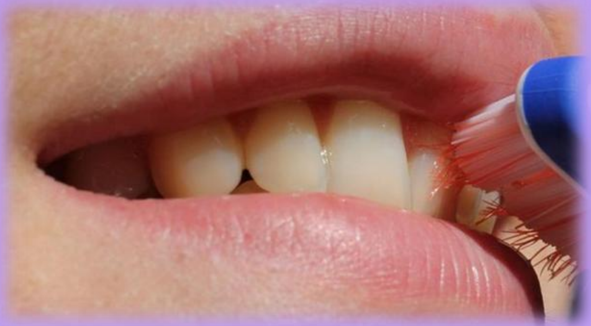

Health of your mouth

During this journey your mouth will undergo lot of changes, especially challenges. Therefore, it is important to enter this journey with a very clean mouth so that during the challenges the discomfort will be minimal.

Due to the discomfort in your mouth, you might tend to neglect your good practices to keep your mouth clean. Please do not give into this. You must pay more attention to your mouth than any other day. This will assure you a successful outcome

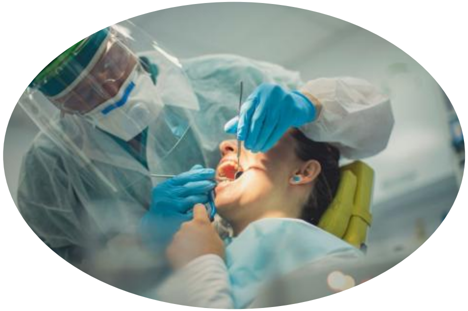

- If you have any decayed teeth, loose teeth or gum disease (bleeding gums, swollen gums, bad breath etc.) treating those problems before the surgery or other treatment, is very important for a speedy recovery.
- Clean your mouth twice daily. You might find difficult to brush your teeth as usual with the tooth brush. You would have severe pain or difficulty in mouth opening. Therefore, initially, try to brush teeth gently, using a juniour tooth brush with a Fluoridated toothpaste. Having soft bristles in the brush is very important.

- If cleaning your mouth is difficult and impossible as mentioned above (since mouth opening is very limited) wrap a clean piece of cotton on a finger or a stick, then soak it in Baking soda & thoroughly clean every surface of the mouth.
- Sometimes your doctor may advise you to use an antiseptic mouthwash for a while. Get the proper instructions for using the mouthwash, from nurses at the Clinic or Ward.

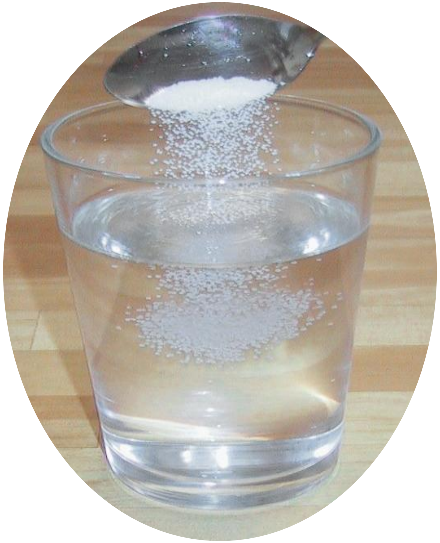

Also, you could use salt water, by mixing one tea spoon of salt into two & half cups of water (600ml) to wash your mouth at home. Take a mouthful of salt water, rinse thoroughly & spit out. Do this 4-5 times per day. Doing this after every meal, will help you in reducing germs, increasing appetite, reducing taste problems, & also to reduce mouth odor.

If you are after radiation therapy, this special mouthwash can be used to reduce the risk of oral Mucositis (the inner surfaces of your mouth is reddened and tender). Add half tsp Salt & one tsp Baking soda into one liter of water (four cups) & use this mixture to wash your mouth several times per day.

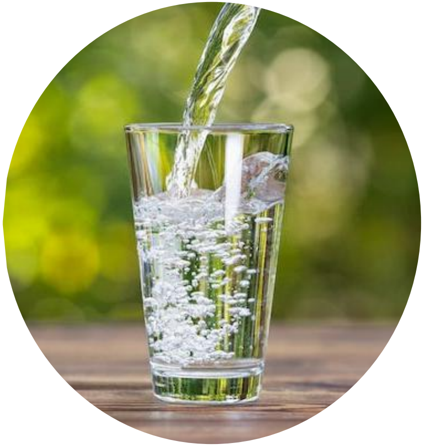

- It's normal to feel dryness of your mouth. Drink ample water frequently to minimize this. Also, if you can afford to buy, you can use the Artificial Salivary products which are available in the pharmacies.
- Reduce sugary food as much as possible. This will help you to protect your teeth as well as, to reduce growth of cancer cells & to control Diabetes.

The latest studies confirm that maintaining good Oral health, despite of all the temporary difficulties, is important to defeat cancer.

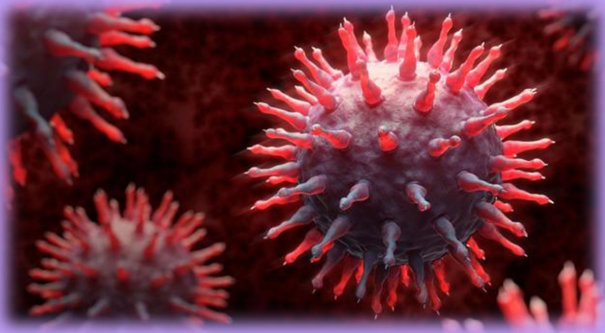

Prevention of Infections (Minimizing Germs)

During this time, your ability to fight against other infectious diseases (germs) is less than before. So try to adhere to the following.

- ✓ Strictly follow all the medical advice.
- ✓ Both the patient and the caregiver need to be clean.
- ✓ You should have a bath/ bodywash daily, if you are free of wheezing and diseases like cold.
- ✓ After bathing, swab & dry the wounds, using a soft clean cloth.
- ✓ The caregiver must wash hands thoroughly before and after handling the patient.
- ✓ Collect the infected waste separately, that is the discarded dressings of wounds, anything that is with blood etc., Put them separately into a bag & bury or give it separately to the Garbage vehicle.
- ✓ If any family member is having an infectious disease, isolate the patient.
- ✓ Keep the patient's surrounding clean.
- ✓ If there are too many insects like flies in patient's living place, have a mosquito net around the patient.
- ✓ Try to avoid being in crowded places. If it is unavoidable then, use a face mask.
- ✓ If you notice fever, cough, diarrhea, redness of the skin or ulcers immediately consult a Doctor.

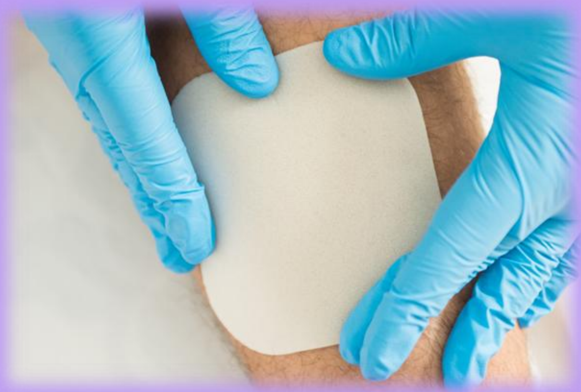

Proper Wound Care

Most probably, your wounds will be largely healed, by the time you go home after the surgery. However, follow the instructions given by the hospital regarding wound care properly.

- If you have been given a mouthwash, use it properly. Get the instructions form the ward or clinic.
- Properly keep to the appointments given by your clinic.
- By any chance if there is bleeding from the wounds, place a sterile gauze firmly (but carefully) on the wound & hold it for a while. However, if bleeding doesn't resolve, meet a doctor as soon as possible.

1. Physical challenges

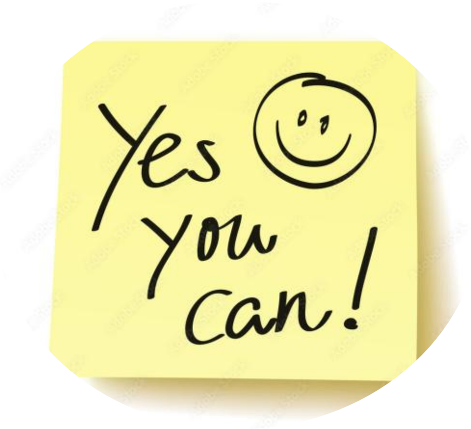

- Majority of the changes in your appearance, following the surgery, are temporary. Therefore, don’t worry.

- Your doctors always try their best to minimize the undesirable effects. Until improvement you can cover the affected area with a clean towel or a face mask, especially when you are out or in your working place.

- If you were addicted to betel chewing (with or without tobacco), arecanut chewing, smoking or alcohol before, stop these habits immediately. You CAN !!
- If you need any help call 1948, using any phone network.

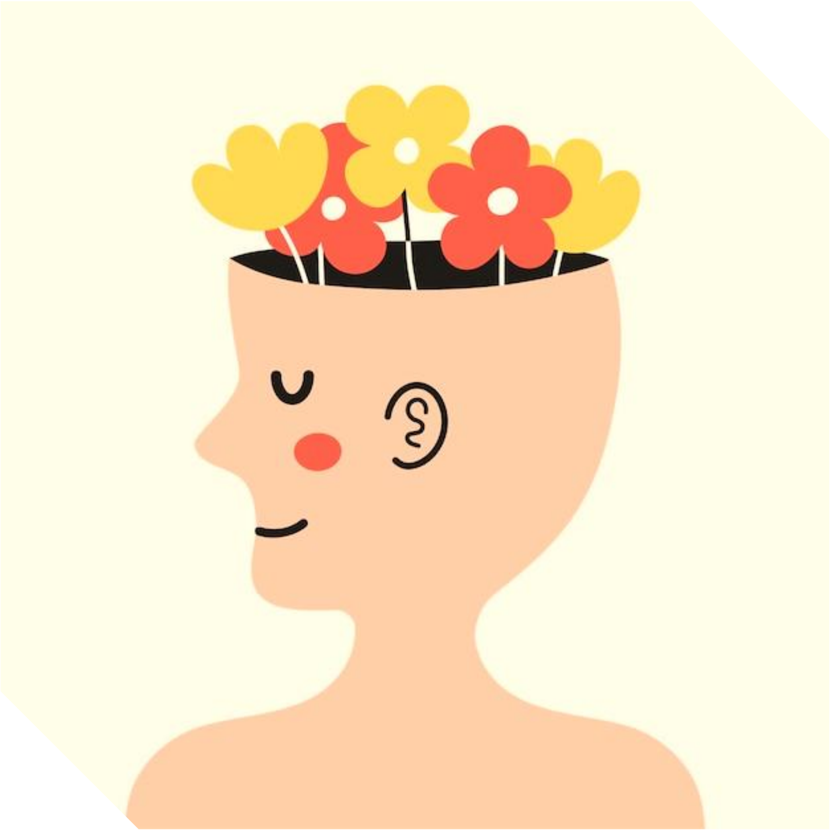

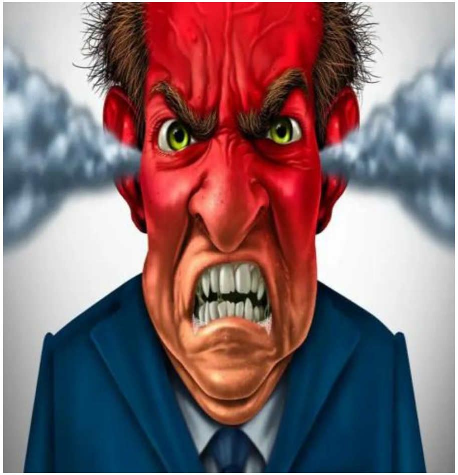

Could you recall a moment when you got angry? What exactly happened to your body?

Your heart rate went up, right? You felt warmth all over your body. You were trembling, weren’t you?

If you properly think about it, anger is a feeling which takes place in your mind. Yet, your whole body was changed. This means your body is directly affected by, your thoughts, emotions- how you feel.

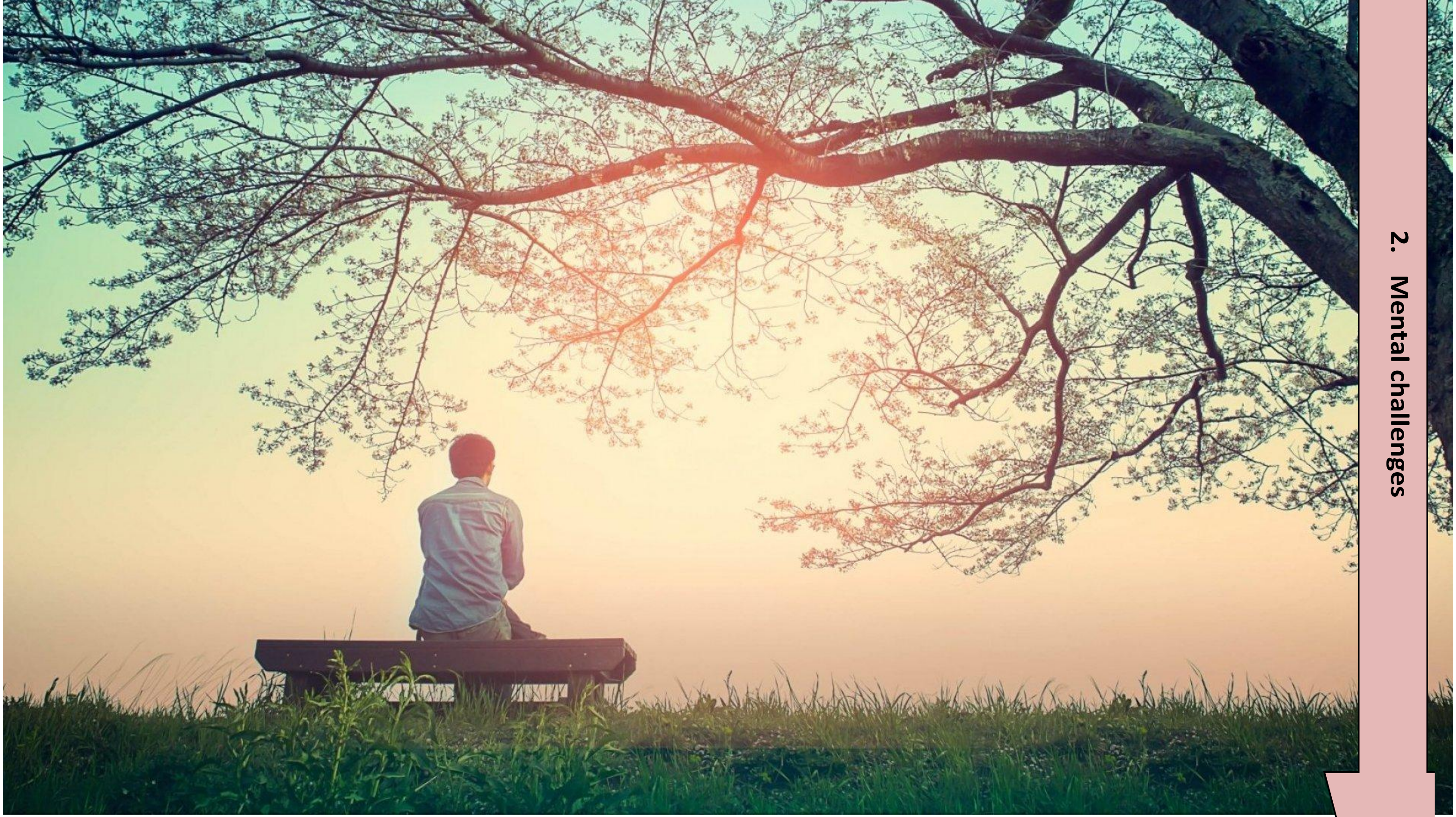

2. Mental challenges

Right now, your body is facing some challenges which is short term. If you want to bring the body back to normal, your mind must be strong. That means you should be happy.. How can anyone be happy with a cancer?

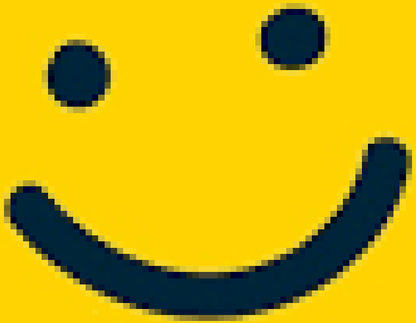

Can you stay happy, when you have a cancer?

Yes, you can...

2. Mental challenges

When you got to know, that you have an oral cancer, it is normal to be sad and worried. So it is alright. Let it be. Accept it. But you don't have to stop there. Because,

- Nowadays cancer treatments are very advanced. There are several methods to cure & control the cancer.
- As was mentioned earlier, your mind controls your body. if you want to recover fast and bring your life back to normal, you must stay happy. It is alright to worry for a moment but do not dwell there. The more you worry, it is less likely for you to recover quickly. So, accept your sadness, worry, disappointment, frustration- It is alright to feel all this. Now let go. move on to your life ahead.

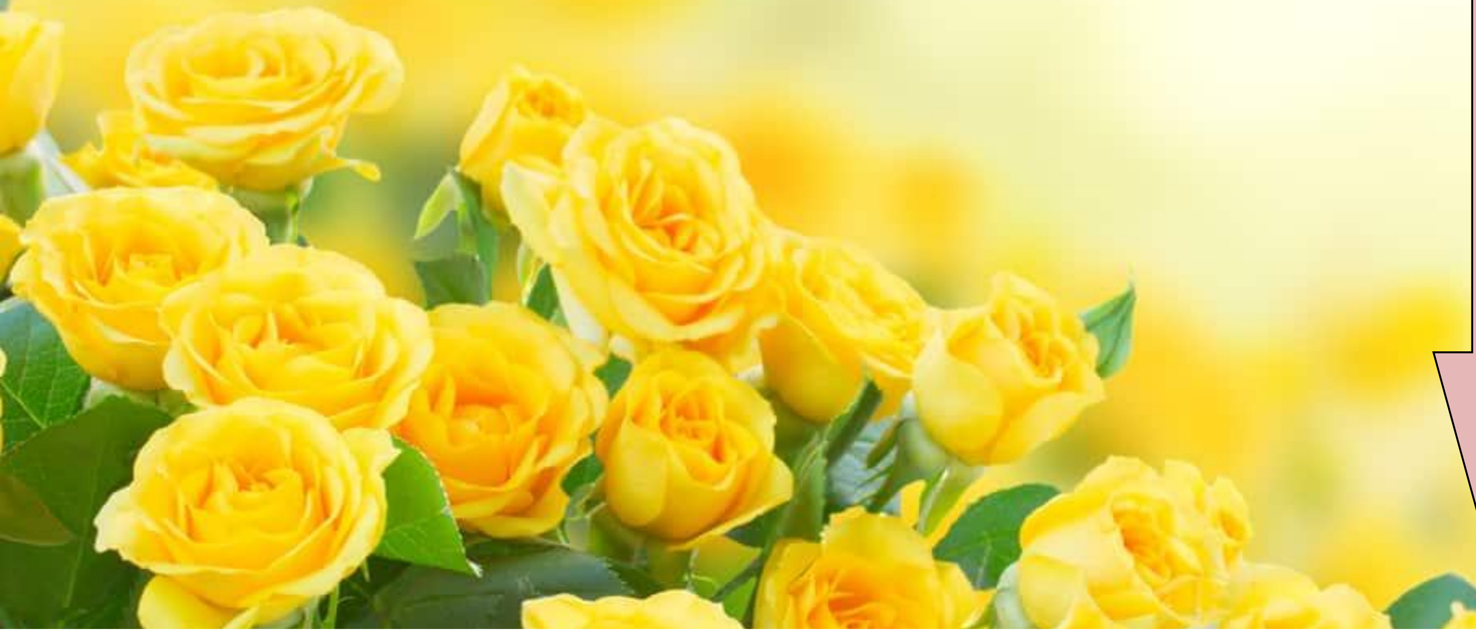

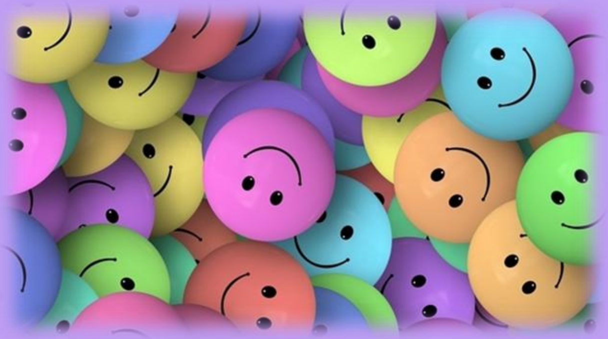

Tips to stay happy

✓ Tip 1 - Live NOW!

All we have is NOW. The easiest way is to live fully in the current moment- NOW. We usually regret, ‘Oh ! In vain…This Disease!! Why it must be me??’ and regret about the things that happened in the past, don’t we? Or we think about the future, ‘What will happen to me? What will happen to my family and loved ones?’ and we worry. Don’t we?

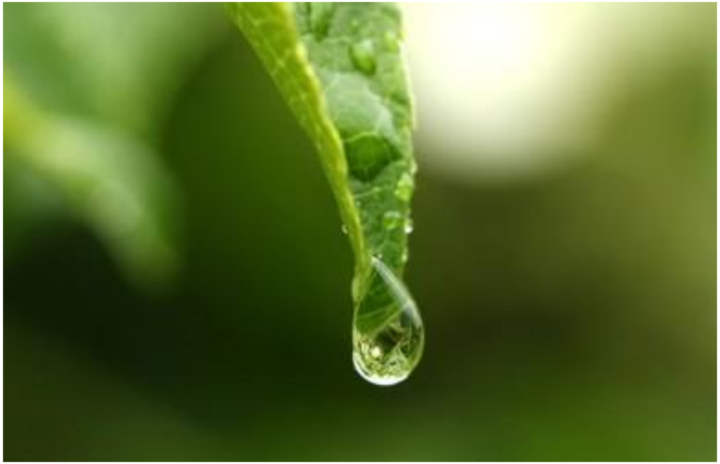

Shall we fully give our attention to this moment ... that is, right NOW... You are reading this paper now and can you do it with your whole self? If your mind goes here & there, bring it right back to this moment...NOW – This is how you do it. When your mind travels away focus on your five senses.

- **LOOK** at who is sitting next to you right now. Look at the book. This book is white with black letters. Look at the pictures in the book.
- **TOUCH** the book pages. What do you feel? Is it smooth or rough. Is the book pages cold or warm?
- **LISTEN** to what you can hear now? Do you hear the people around you? Vehicle noise? Birds. Just keep on listening.
- **FEEL** – What your body feels now? Is it cold? Warm?

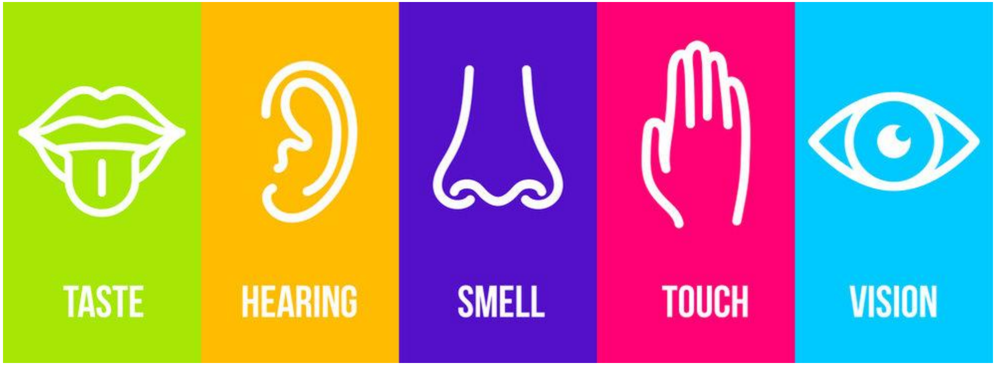

- In this manner, carry out whatever you do with your whole self. Try it for one day.
- Start now. All the daily activities you do, carry out mindfully. When your mind travels away which is normal, bring it back by looking at something near you, listening, smelling and feeling. You will feel the difference and the relief.
- Yes, initially you might find little difficult but as you continue to do this your mind learns to be fully present in this moment. Remember this will help your body to fight very well with cancer.

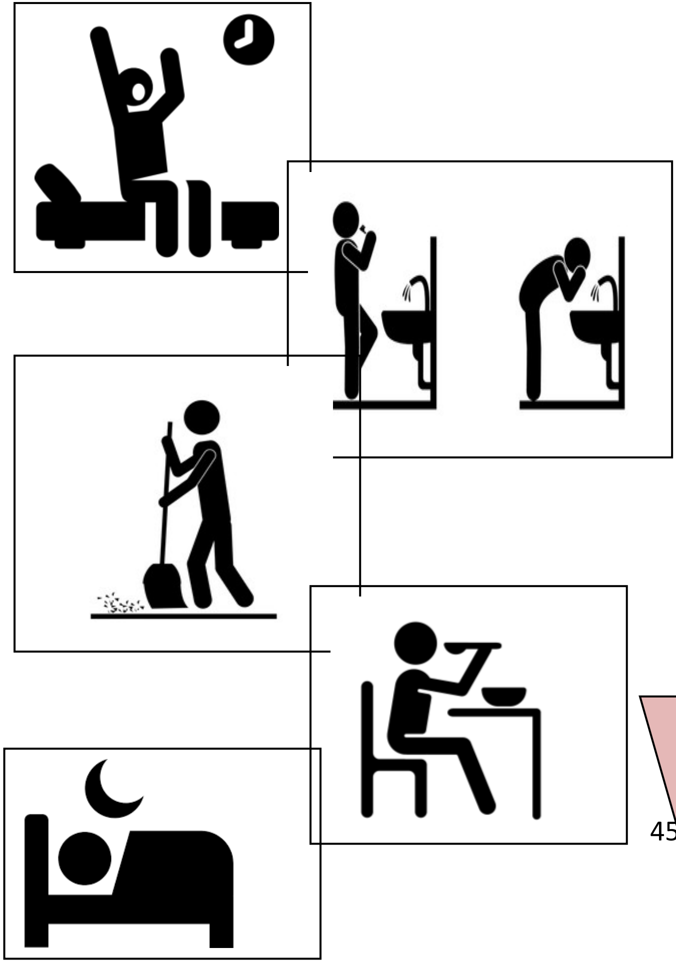

Perhaps right now your thoughts might be focused only on your disease, which is alright for a moment. But let's move on.

Life brings challenges continuously for some it's a disease like cancer, for some a financial struggle and for some a relationship issue. What really matters is not the nature of the problem but how we respond. So, let's respond to this challenge correctly. This will make your life beautiful.

Remember, life is beautiful.

- ✓ Tip 2- Have your normal life

Cancer is a disease that anyone can get. Therefore, move about happily with your children, relatives, and friends as you used to do. Be joyful! Try your best to spend life in the same way you have got used to.

- ✓ Tip 3 – Get involved in doing what you like

Get involved in your hobbies more. Read newspapers and books. Take time to watch and enjoy television. Listen to music. Engage in new hobbies that keep you happy!

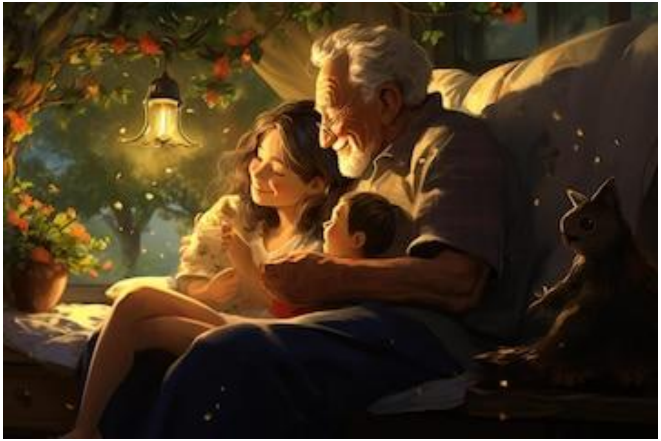

- ✓ Tip 4 – Ask for help!

If you have any problem, concern, which worries you & which you can't share with your family, there are lot of people who is willing and capable of help you.

- Counselling Unit (Apeksha Hospital, Maharagama) – 011 2844459
- Palliative Care Unit (Apeksha Hospital, Maharagama) – 011 2850252
- Health Education Unit (Apeksha Hospital, Maharagama) – 011 284 2052
- 'Sahan Suwa' - 011 7600444
- Indira Cancer Trust - 011 2363211
- Hot Line-National Institute of Mental Health -1926
- Counselling services at your Divisional Secretariat

If you feel anxious or stressful continuously, always get specialized medical advice.

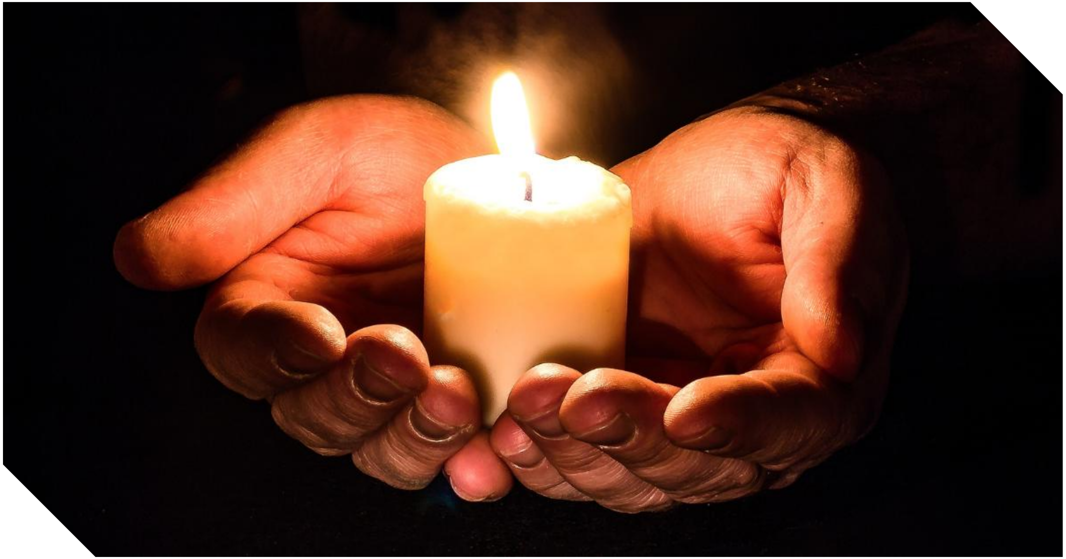

3. Spiritual challenges

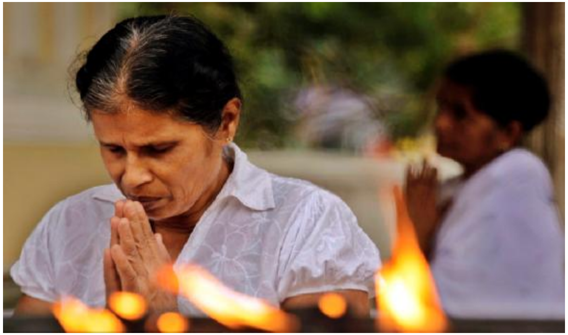

- If you are used to visit temples/ churches/ mosques, continue to engage in those activities. It’s better, if you can spend more time for this.

- Engage in more religious activities at home too. Have a picture of your religious leader, in a visible place in your room. Get involved in meditation, Bodhi Puja etc. if you are interested in them. This will make you happy and give a better outcome.

- Try to listen/watch the religious programmes, which are being broadcasted in Radio, TV.

- How are you feeling now? You could be someone who believed in your religion / philosophy and now are disappointed. This can happen to anybody. Meet the clergy in your village or who are close to you and discuss about this with them.

- Perhaps you could be a person who didn't believe in a religion / philosophy. Yet, you might have had a meaning to life… some vision in life, goals in life? Isn't it? Now, what do you think about life after being sick? ... Give a thought to these. If you feel lost or helpless, you can get help from a trustworthy friend or from the places mentioned above.

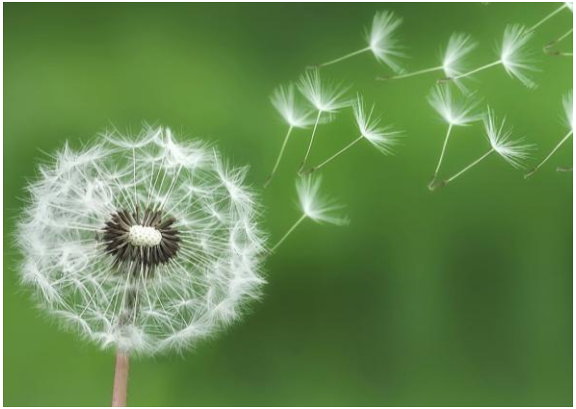

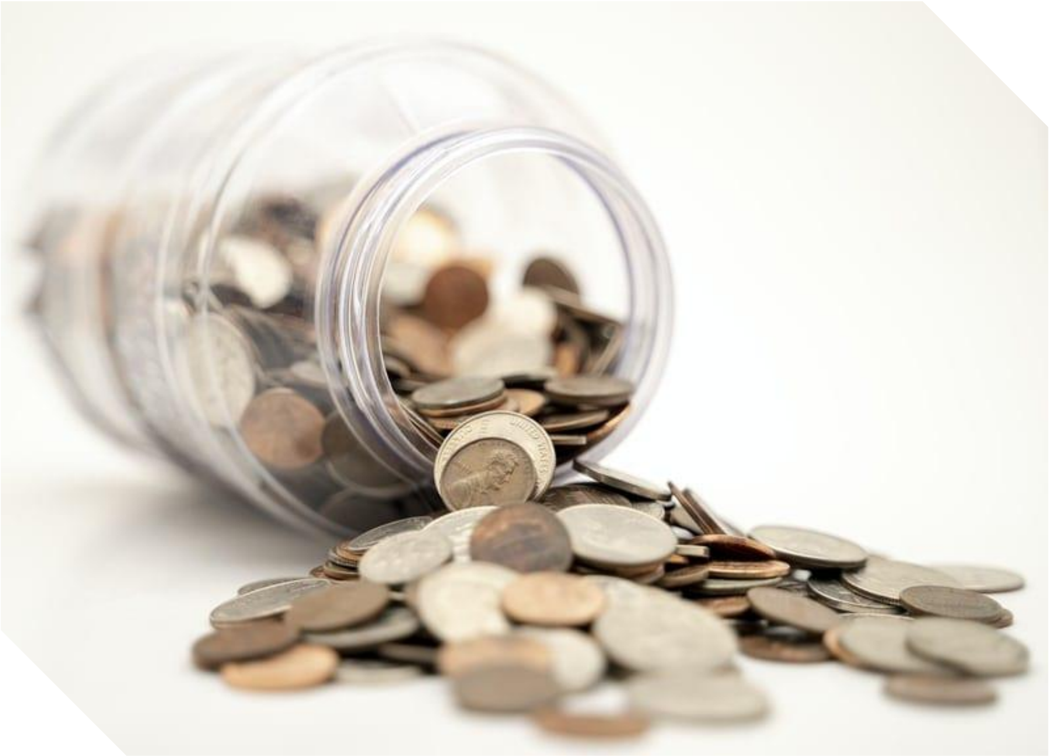

4. Financial challenges

• Try your best to continue with your current job. Explaining about your condition to the administrative officials at your workplace will help you to get some relief.

• If you find it difficult to continue with your current job due to the illness, or if you are not employed at present, you can engage in a self-employment. The instructions, guidance and training for self–employment could be received from Counselling Unit (Apeksha Hospital, Maharagama) – 011 2844459.

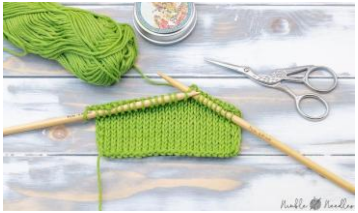

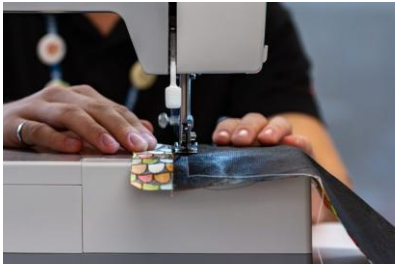

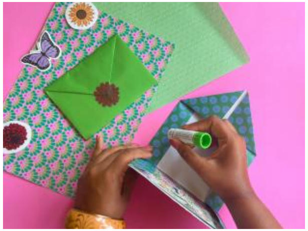

- Also, you can get an allowance from your Divisional Secretariat. Further information regarding this can be obtained from your clinic or the Divisional Secretariat on every Wednesday.

- You may have relatives or friends who can help you during this challenging time. Seek help from them.

- The following places would be able to help you with some relief.

- Sri Lanka Cancer Society (Only for the patients at Apeksha Hospital) - 0112585879
- Indira Cancer Trust - 011 2363211
- 'Mithuru Wala' Supportive Network - 011 2854325
- Cancer Association - 011 3155229
- 'Sahan Suwa' - 011 7600444
- 'Prashakthi' Women Organization (Only for the patients at Apeksha Hospital) 011 2844383/ 072 8824889/ 011 2509102/ 0711208197)
- 'Sathya Sai Suwa Sevana' - 036225490

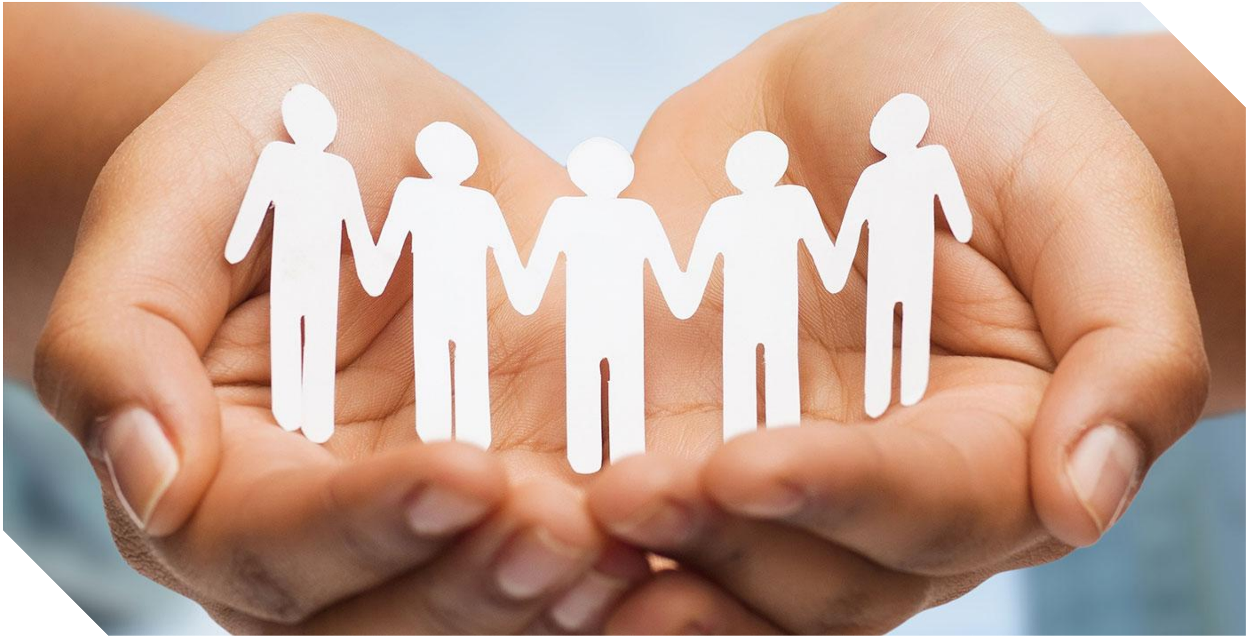

6. Social challenges

This is about you connecting to people around you. Remember that all around you, your loved ones are going through a challenging time as you are. So they might behave different at times. Have patience with them. Accept them as they are. Always keep in mind that they love you and doing everything they can to help you through the challenges.

- ✓ Spend more time with them
- ✓ Talk to them and listen to them
- ✓ Enjoy everything you do with them
- ✓ Laugh together!
- ✓ Make beautiful memories with them

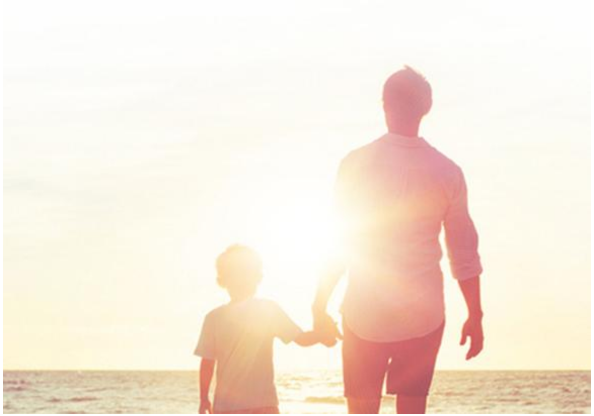

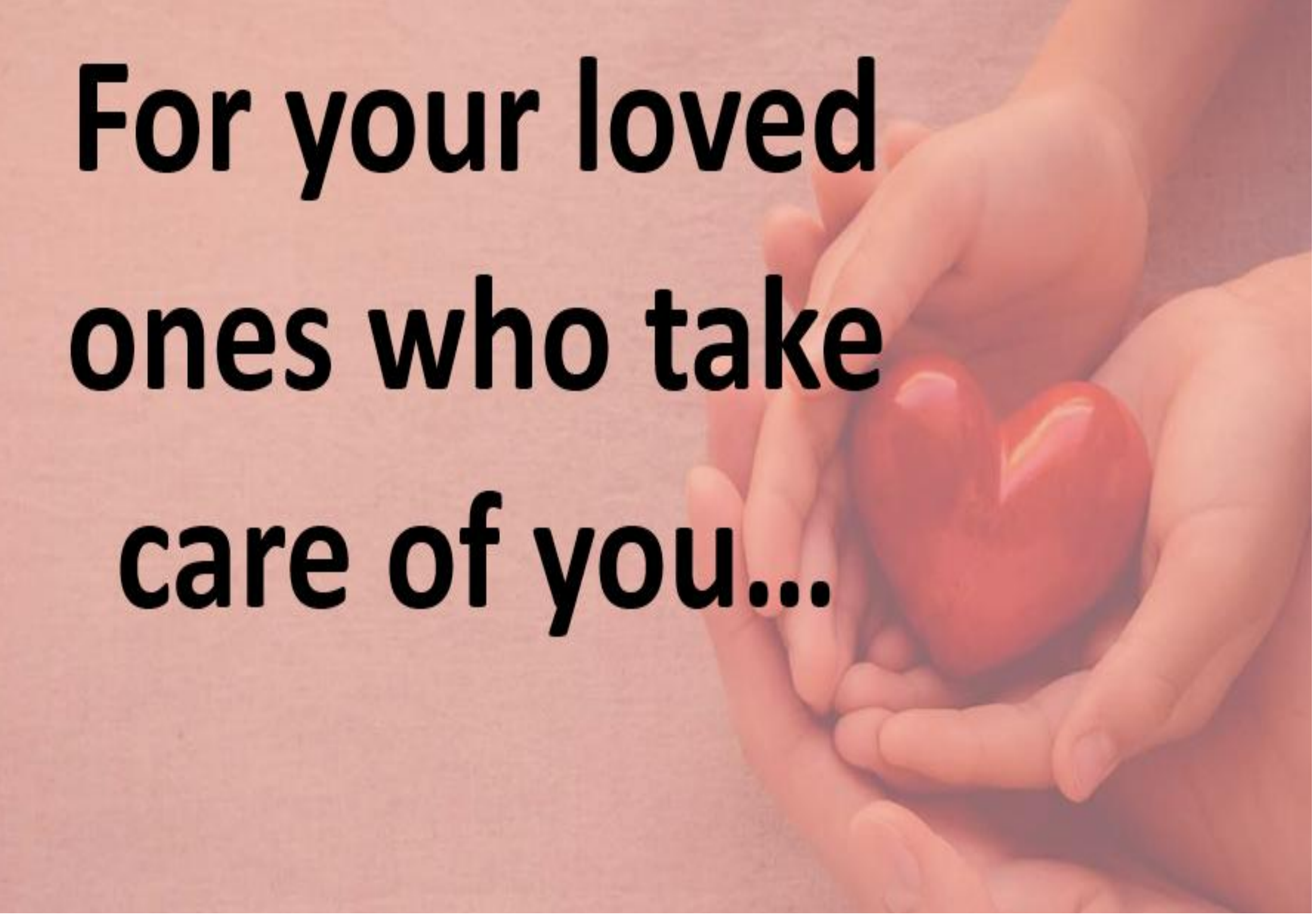
For your loved
ones who take
care of you...

- This commitment of yours, is a great charity, which gives you a lot of happiness & comfort. Therefore, even if you feel tired at times, do it happily. Your help is very much needed for your loved one to recover fast.
- Depending on the situation your patient is going through, he/she might get angry easily or would not interact with you much. Be patient and bear up these happily. These will not last for long for sure.
- Talk with the patient, whenever you can. Stay close to him/ her. Listen. Be kind,commpasionate, gentle, caring and sharing.

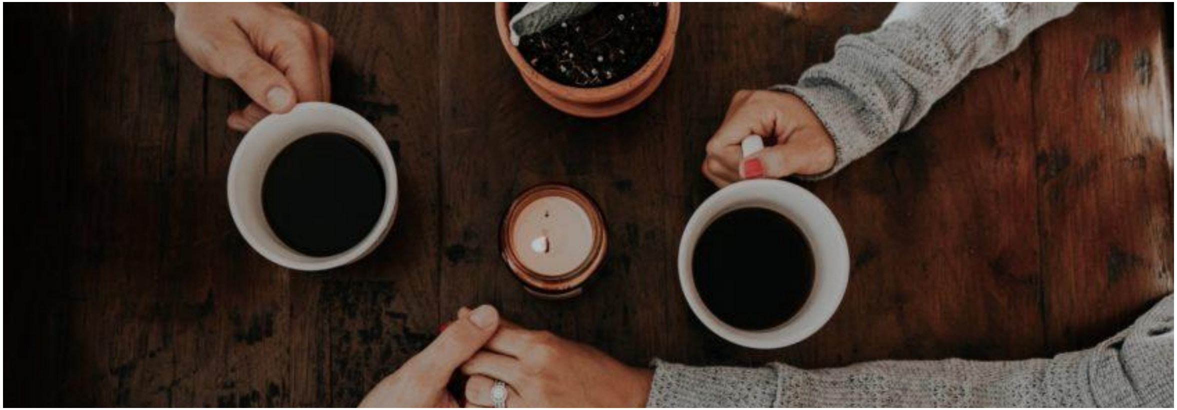

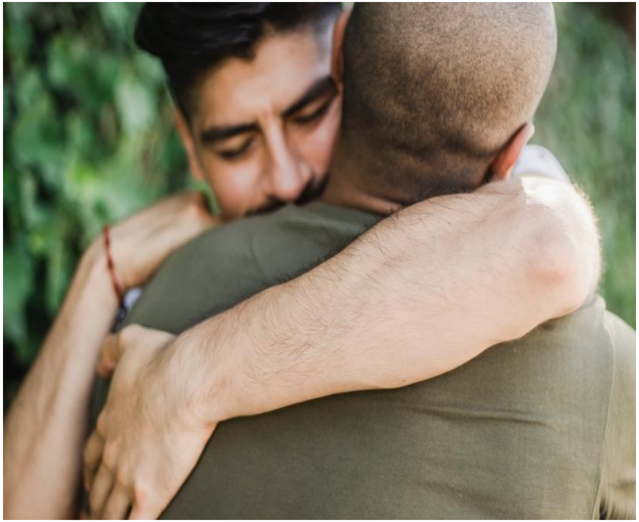

Help your loved one to lead a life as normal as possible. Do not force him/her to always stay in bed, thinking they are sick. Let them do their day-to-day work after getting doctor’s approval. This will keep them happy and occupied, that help them to recover fast.

During the treatments, your loved one might face physical problems as well as problems in their minds. There may be changes in their appearance as well. All these are very temporary. Always remind this to the patient. And you too keep these in mind and stay strong!

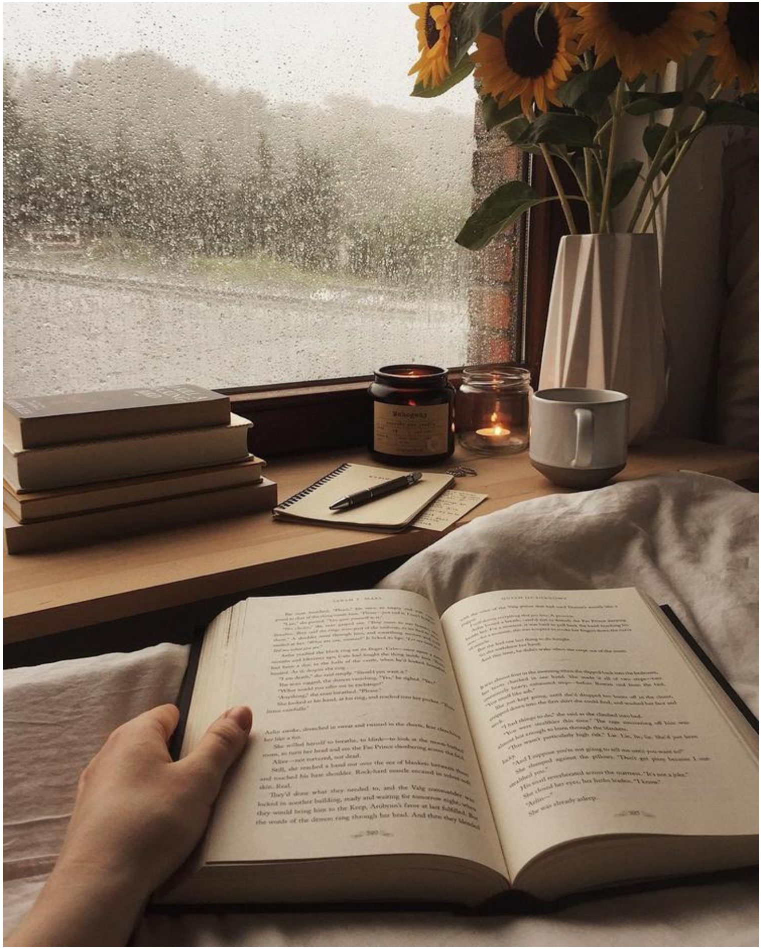

In the same manner you take care of your loved one, take care of yourself too. Take a short break. Spend some time doing whatever you like.

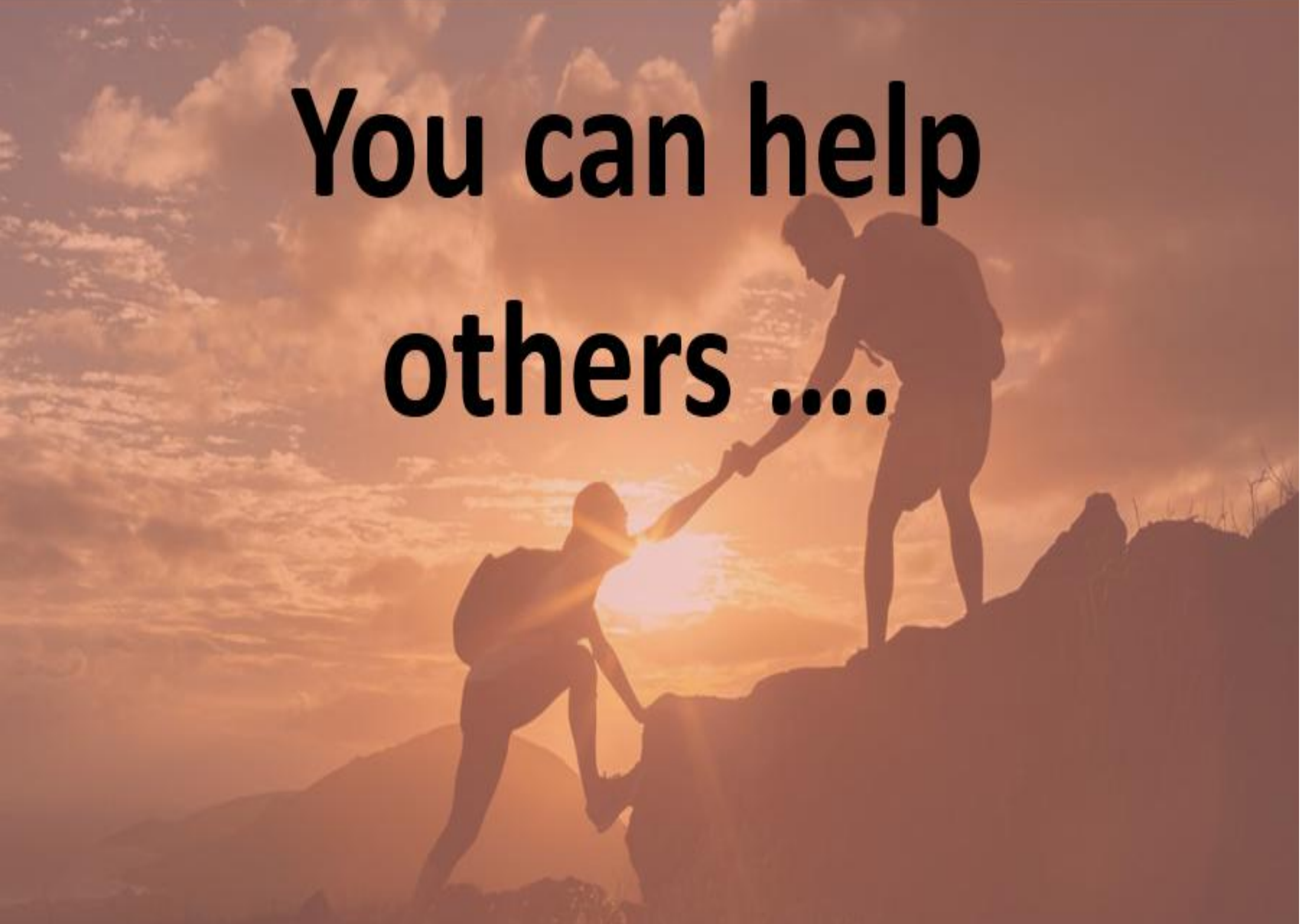
You can help
others ....

Now you are certain that you can overcome a lot of difficulties, by being happy, following your doctor’s instructions and the tips in this booklet. Will that be enough? Are you going to stop there?

How great it would be, if you can go beyond that & give a valuable message to the society, from what you have gone through? You can give a valuable message, to prevent another person going through this. Isn’t that a great charity?

- Make your friends and relations aware of this cancer. Read the annexure attached to the end of this booklet. Use this knowledge to educate others. Save them from the habits that causes oral cancer. You can do a great service, especially to save the youth from bad habits. Tell your friends and relations around about the common reasons for mouth cancer and help them to stop the habits and go for check ups.

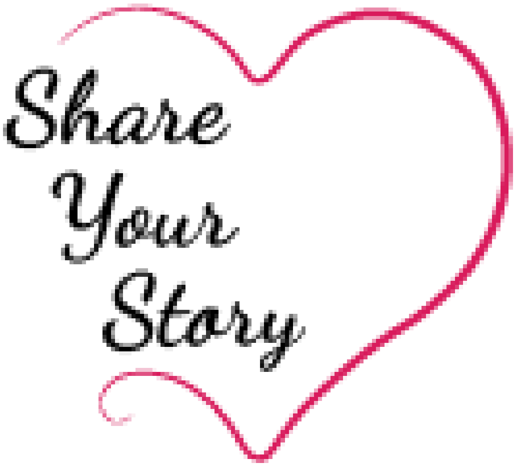

- Similarly, you can share your experiences to give strength to another cancer patient to go through this challenge. You can do a lot to reduce their fear by sharing your success story.
- The message you give to society is much more valuable and effective than it is said by a doctor or a health official.

- Do you know? Sri Lanka has an organization called ***"Nil Katarolu Niwahana",*** which is formed by the patients who have mouth cancer. You could serve society through them as well. Contact them on 0112- 888061.

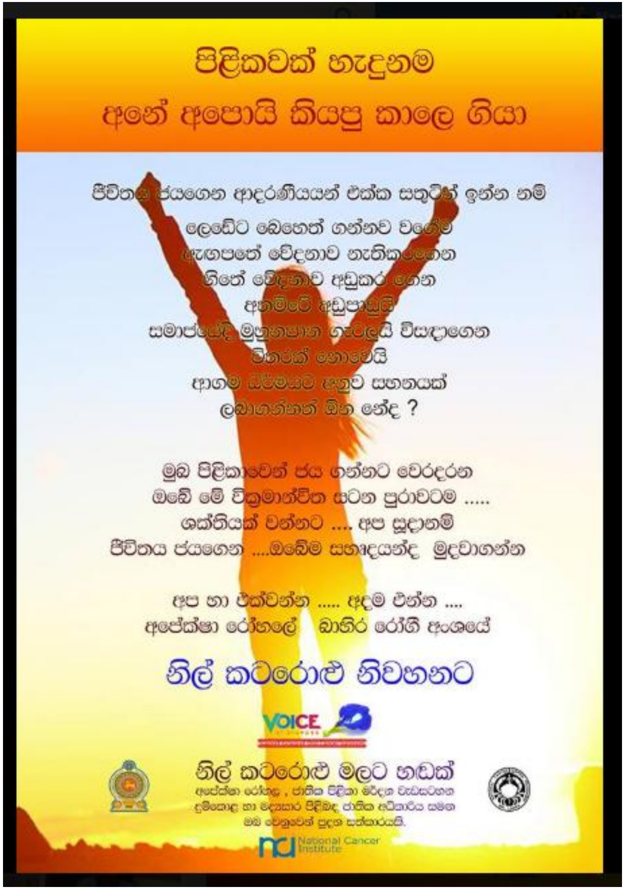

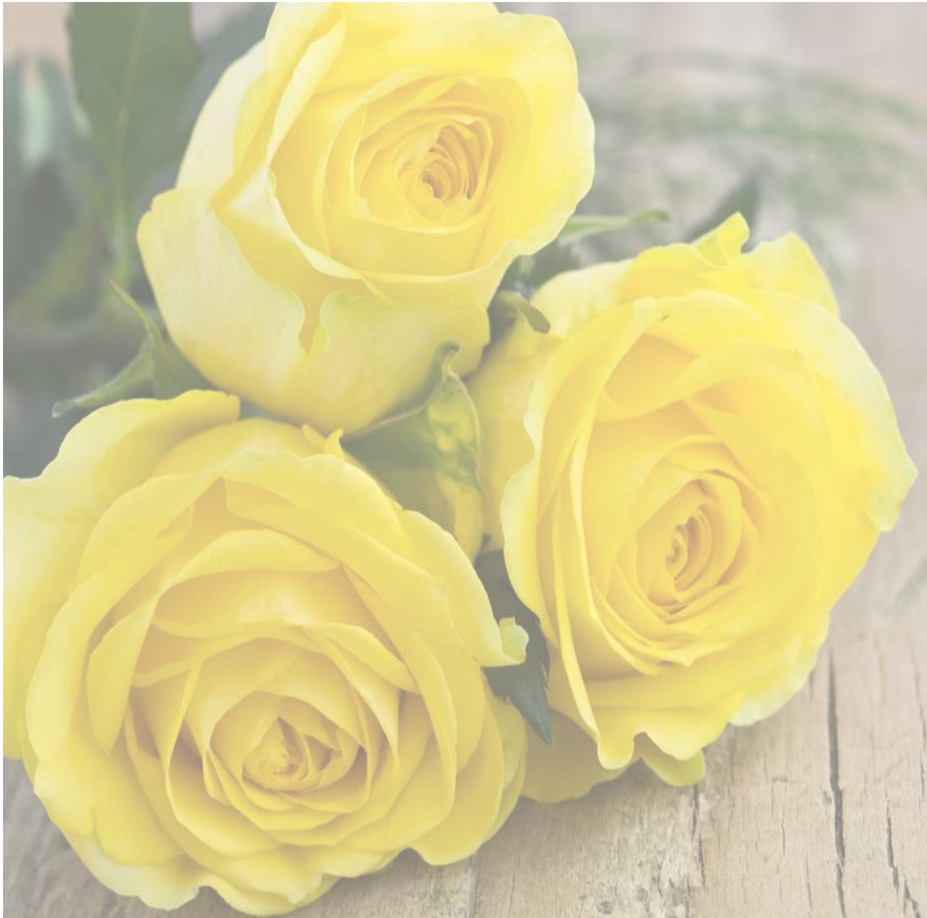

You can definitely fight cancer to become a winner…

May you get well soon……!!

The booklet was designed by,

- **Referring research carried out in the World and in Sri Lanka to date.**,
- **Valuable inputs from experts in Oral & Maxillofacial Surgery, Oncosurgery, Community Dentistry, Community Medicine, Psychiatry & Sociology**
- **Valuable inputs from patients with oral cancer & their caregivers**
- **Using papers & books, published by Apeksha Hospital, Maharagama & National Cancer Control Programme Sri Lanka.**

The expert committee

Dr. Padma Silva – Consultant in Oral & Maxillofacial Surgery

Dr. D. K. Dias - Consultant in Oral & Maxillofacial Surgery

Dr. S.P. Kandapolaarachchige – Consultant Psychiatrist

Dr. Suraj Perera – Consultant in Community Medicine

Dr. Kanishka De Silva – Consultant Onco-surgery

Dr. Prasanna Jayasekara - Consultant in Community Dentistry

Dr. Irosha Perera - Consultant in Community Dentistry

Dr. Hemantha Amarasinghe - Consultant in Community Dentistry

Dr. Sharika Gunatilaka - Consultant in Oral & Maxillofacial Surgery

Dr. Jayamal De Silva - Consultant Psychiatrist

Coordinator

Dr. Nadisha Ratnasekera

Annexure

The truth about oral cancer?

Oral (mouth) cancer is the commenest cancer among the Sri Lankan males. Although earlier it was mostly among elderly, now, the youngers are also being affected. The majority of the oral cancers are preventable.

- What is cancer?

  Cancer is the uncontrolled growth of abnormal cells in the body. We all have cancer cells in our body. Our own immunity controls these cancer cells. However, when this control is interfered with other surrounding factors, it can lead to cancer.

- How to prevent mouth cancer?

- ✓ Following habbits should be stopped.
  - Betel chewing (Arecanut, Tobacco, Lime)
  - Arecanut packet usage (young children are prone to use these.)
  - Smoking
  - Alcohol use
- ✓ Additionally, you should eat food, that enhance your immune system.
  - Fresh fruits & vegetables. (at least five types per day.)

    - Tomato, Garlic, Cardamone, Nelli, Rasakinda.
- ✓ It is important to maintain good oral health and healthy gums.
- ✓ Be physically active – exercise at least 30 minutes a day

- ➢ <u>How to detect mouth cancer or a condition which can lead to mouth cancer.</u>
    - Red/White spots inside the mouth which is present for more than two weeks.
    - Non healing ulcer/s in mouth for more than two weeks.
    - Enlargement of Lymph nodes around face & neck.
    - Mouth opening difficulties.

Make it a practice to check your mouth for presence of any of the above features by standing in-front of a mirror. If any of the above features are to be seen or if there is any doubt, visit the nearest Dental Surgeon.

Annexure b

The training plan for the two sessions of the training programme for Public Health Nursing Officers

❖ Training plan – session 1- **(To All Public Health Nursing Officers)**

Training cycle of the programme

1. **Assessing and analyzing the need for the programme**

↓

2. **Designing and developing the programme**

↓

3. **Implementing the training programme**

↓

4. **Evaluating of the training programme**

1. Assessment and analyzing the need for the

A- Situational analysis of the organizational needs

One of the objectives of The National Cancer Prevention and Control Policy in Sri Lanka was stated: "To ensure rehabilitation, survivorship and palliative care facilities for patients with cancer and their caregivers at all levels". The second strategy of this objective is to *develop human resources for the delivery of rehabilitation, survivorship, and palliative care services* at institutional and community levels.

Accordingly, the 1st batch of Public Health Nursing Officers (PHNOs) were recruited in January 2018 and after a training period of 6 months, currently they have been attached to Healthy Lifestyle

Centres island-wide. Although basic training was given to them, a continuous capacity-building process was of utmost importance to get the expected outcome of these Public Health Nursing Officers.

In Sri Lanka, the most common cancer among males is oral cancer and it is an all-time public health problem. Furthermore, these oral cancer victims are from low socioeconomic classes and the psychological distress affects adversely on the treatment outcomes of the patients.

B- Analyze the job description of the Public Health Nursing Officers

The Public Health Nursing Officers job description includes a palliative care module which was summarized in table 2.3 at the literature review.

Summary of core areas identified as training needs in palliative care for patients with oral cancer within the purview of service provision of Public Health Nursing Officers

- Epidemiology of oral cancer in Sri Lanka
- The Concept of palliative care
- Management of patients' acute issues – pain management, wound care, rehabilitation of basic functions etc.
- Nutrition care of the patient
- Basic psychological support
- Mindfulness therapy
- Habit intervention

2. Designing and developing the programme

- **Training Objectives**

To enhance the capacity of the Public Health Nursing Officers to provide palliative care for patients with oral cancer who were just after surgery which would improve the psychological distress and the quality of life of the patient

- **Content identified for the training programme**

1. Epidemiology of oral cancer in Sri Lanka and identify it as a public health burden
   - A lecture note was provided
2. Refresh/ update the knowledge on palliative care
   - A lecture note was be provided
3. Management of patients' acute problems –pain, wound care, rehabilitation of basic functions etc.
   - Lecture notes was be provided
   - A tool to assess 'pain' was introduced
4. Nutrition care of the patient (special focus on after surgery period)
   - Lectures were done based on a standard diet plan which was in practice at National Hospital Sri Lanka
   - Methods of monitoring the nutrition of the patient after surgery was done.
5. Basic psychological support (Ventilation of emotions, providing Information and education, problem solving counseling and maintaining hope)
   - A comprehensive note was given
   - A standard book prepared for health care workers on basic counselling skills was provided
   - Role play and group discussions was used
6. Mindfulness therapy
   - Introductory lecture on Mindfulness therapy

- Few practical sessions on mindfulness therapy was done

7. Habit intervention
   - Lectures and case scenario was used
   - A standard book on habit intervention was provided

- **Training methods**
  - ✓ Lectures
  - ✓ case scenario
  - ✓ Group work
  - ✓ Role play

- **Training Materials**
  - ✓ Power Point presentations
  - ✓ Printed notes
  - ✓ Booklets
    - "හැම අඳුරු වළාවකම රිදී රේඛාවක් ඇත…. හැම අඳුරකටම එළියක් ඇත…." The book developed by the PI for the current study
    - 'Basic Counselling skills for Health Professionals'
    - A handbook on habit intervention
  - ✓ leaflets
  - ✓ Case scenarios
  - ✓ Video clips

- **Resource persons**
  - [redacted] – Consultant in Community Dentistry- Preventive Oral Health Unit- National Dental Hospital (Teaching) Sri Lanka
  - [redacted] – Consultant OMF Surgeon- NDHSL
  - [redacted] Consultant Surgeon oncology, Apeksha Hospital
  - [redacted] – Consultant Psychiatrist – Apeksha Hospital
  - [redacted] – Consultant Community Physician, National Cancer Control Programme

- [redacted] – Consultant in Community Dentistry, National Cancer Control Programme
- [redacted] Consultant OMF Surgeon, Karapitiya Teaching Hospital
- [redacted]- Consultant Anesthetist, National Hospital Sri Lanka
- [redacted] - Consultant in Community Dentistry, IOH Maharagama
- [redacted]- Nutritionist, National Hospital Sri Lanka

The fact that the delivery of the intervention package was highly dependent on the nature of the training given as well as the Public Health Nursing Officers' attitudes and skills were acknowledged throughout. Therefore, this was emphasized to the resource persons of the training programme and all possible measures were taken to provide a comprehensive and more practical training programme.

3. Implementation of the training programme

- ✓ No of days – 2 days (1st and 2nd August 2019)
- ✓ Time: 8.30 – 3.30pm
- ✓ Venue: Post Basic Nursing College, Colombo
- ✓ Financial support: ET&R, Ministry of Health
- ✓ Organized and coordinated by – Dr. Nadisha Ratnasekera – Registrar in Community Dentistry
- ✓ Supervised by – [redacted] – Consultant in Community Dentistry, NDHSL and Dr. [redacted] - Consultant Psychiatrist, Apeksha Hospital

4. Evaluation of the training programme

The process used for evaluation of the training programme is summarized in table 3.7.

Table 3.7 Training Evaluation Steps According to Kirkpatric Training Evaluation Model

Level	Description	Method	Monitoring Indicators	Time period
Reaction	Did they like the programme?	A question on satisfaction was included in the post assessment form (annex XLII)	- % of participants who were satisfied with the training programme	At the end of the programme
Learning	Did they learn it?	Pre-test questionnaire (annex XLI) post-test questionnaire (annex XLII)	The mean knowledge score difference of PHNOs	At the beginning of the programme and at the end.
Behavior	Do they use it?	22 PHNOs were closely monitored for their performance (these 22 PHNOs carried out the intervention for the research)	- No. of patients who received palliative care<br>- No. of home visits paid	After 2 months of the training programme
Results	Did the training improve the results?	The patients' psychological distress and the quality of life on whom the intervention was carried out will be evaluated	- Analysis of pre and post-intervention psychological distress of the patient<br>- Analysis of pre and post-intervention patients' quality of life<br>- Assessment of the satisfaction of the patients	-Pre-intervention<br>-Just after the intervention<br>-3 months after the intervention

- Training Plan session II - **(For 22 Public Health Nursing Officers who were included to carry out the novel intervention)**

Training cycle of the programme

1. Assess and analyze the need for the programme

↓

2. Design and develop the programme

↓

3. Implementation of the training programme

↓

4. Evaluation of the training programme

1. Assess and analyze the need for the programme

a) <u>Situational analysis of the organizational needs</u>

All the Public Health Nursing Officers were exposed to a 2-day capacity building programme. The intervention of the study was planned to be carried out 2 months following the first capacity-building programme. Although all the Public Health Nursing Officers were trained at the capacity building programme, only 22 Public Health Nursing Officers was recruited to carry out the intervention for the study purpose.

Hence it was needed to fine-tune the training programme from the baseline level to build the capacity of Public Health Nursing Officers to deliver the core components of the planned psychosocial intervention efficiently and effectively.

In order to find out the existing knowledge of the Public Health Nursing Officers with regard to palliative care for patients with oral cancer, an electronic mail was circulated to be filled by the Public Health Nursing Officers.

Summary of major areas identified as specific training needs for the selected 22 Public Health Nursing Officers on palliative care for patients with oral cancer

More specific guidelines were required as to how exactly the intervention should be carried out.

2. Design and develop the programme

- **Training Objectives**

To enhance the capacity of the selected 22 Public Health Nursing Officers to provide specific palliative care for patients with oral cancer who were just after surgery which improved the psychological distress and the quality of life of the patient

- **Content identified for the training programme**

To improve the capacity on implementing the novel intervention package

a. A brief note on the importance of psychological distress and the quality of life of patients with oral cancer
b. Details of the novel intervention package
c. Instructions on how each component of the novel intervention is to be carried out
d. Administrative procedures

- **Training methods**
  - ✓ Contacted the relevant Public Health Nursing Officer personally and the specific training was done through a web-based video call.
  - ✓ If there was a need PI visited the relevant Public Health Nursing Officer

- **Training Materials**
  - ✓ A handbook
  - ✓ A video clip

- **Resource persons**
  - o Dr. Nadisha Ratnasekera- Principal Investigator
    (If and when the need arose the experts who contributed to the previous capacity-building programme was contacted)

3. Implementation of the training programme II

- No of days – 1 day
- Time duration: 5 hours
- Organized and coordinated by –

  [redacted] – Registrar in Community Dentistry

- Supervised by –

  [redacted] – Consultant in Community Dentistry, NDHSL

[redacted] - Consultant Psychiatrist, Apeksha Hospital

4. Evaluation of the training programme

The process used for evaluation of the training programme is summarized in table 3.8

Table 3.8 Training Evaluation Steps According to Kirkpatric Training Evaluation Model

Level	Description	Method	Monitoring Indicators	Time period
**Reaction**	Did they like the programme?	A question on satisfaction was included in the post assessment form	- % of participants who were satisfied with the training programme	At the end of the programme
**Learning**	Did they learn it?	Pre-test questionnaire<br>post-test questionnaire	Analysis of pre and post total scores gained for the questionnaire	At the beginning of the programme and at the end.
**Behavior**	Do they use it?	30 Public Health Nursing Officers will be closely monitored for their performance (these 30 Public Health Nursing Officers will be the once who will carry out the intervention for the research)	- No. of cancer patients who received palliative care<br>- No. of home visits paid	After 2 months of the training programme

**Results**	Did the training improve the results?	The psychological distress and the patients' quality of life on whom the intervention was carried out will be evaluated	- Analysis of pre and post-intervention psychological distress of the patient<br>- Analysis of pre and post intervention quality of life of the patient<br>- Assessment of the satisfaction of the patients	-Pre intervention<br>-Just after the intervention<br>-3 months after the intervention

Annexure c

Handbook on
A 'life situation improving intervention' for Patients with oral cancer For Public Health Nursing Officers

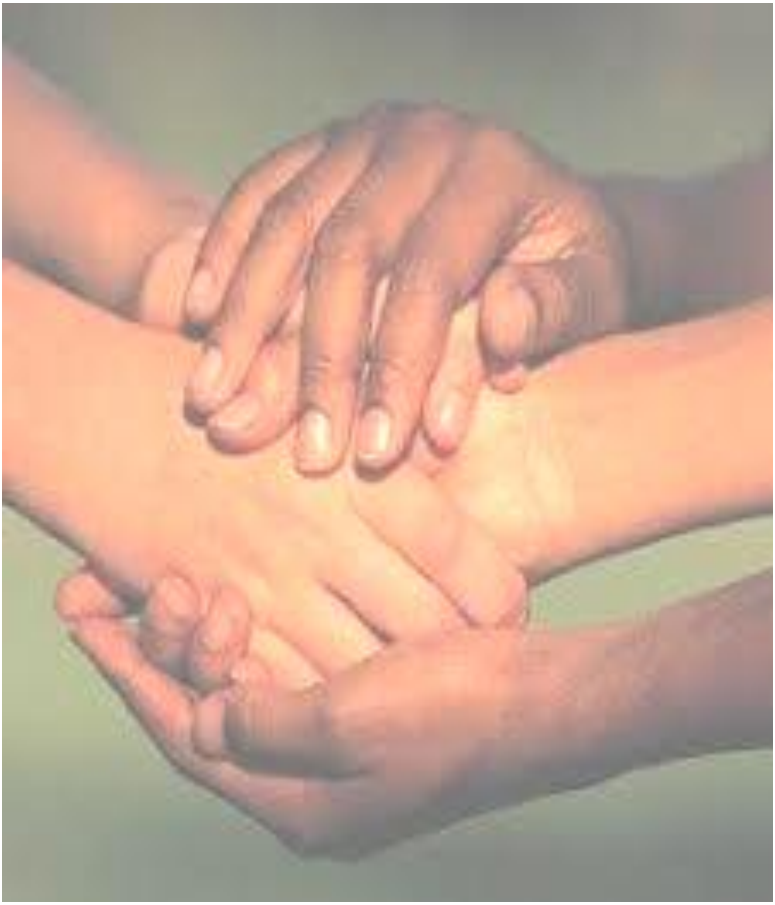

Dr. Nadisha Ratnasekera

Registrar in Community Dentistry

- ✓ **What is ‘life situation’?**

- Combined status of psychological distress (PD) and Quality of Life (QoL)
- Improving ‘life situation’ means reducing psychological distress and increasing the QoL

- ✓ **What happens when the Psychological Destress is high in a patient with cancer?**

- Less compliance to medical treatments
- Slow healing (recovery) from the illness
- Reduced QoL
- More cost to health system

- ✓ **Why ‘Oral Cancer’ is important?**

- The most common cancer among males in Sri Lanka
- Very high psychological distress because,
    - Basic human needs are disturbed. Eg. Speech, eating, appearance etc.
    - Oral cancer commonest among the lowest socio- economic groups. Eg. Farmers, drivers, security officers, estate workers etc. Therefore, their problems are more.

- ✓ **What is the aim of this intervention package?**

  To reduce psychological distress of patients with oral cancer who are waiting for surgery and by doing this to improve their quality of life.

✓ **For whom is the intervention?**

The selection of patients will be done at the tertiary care hospital according to the following criteria. This will be done by the Principal Investigator and the relevant PHNO will be informed about the patient and handed over for the rest of the intervention.

The new intervention will be given to patients who,

- know the diagnosis
- are waiting for surgery as the 1st treatment modality
- have at least one caregiver (married and having children)
- are found to have psychological distress after screening with the Distress Thermometer

‘The Life Situation Improving Intervention’

A diagrammatic pr

**Session number**	**At which point of the treatment journey the intervention is provided**	**Where is the intervention provided?**	**Who provides the intervention**	**Content of the session**
Session one	At the ward prior to surgery	Apeksha Hospital, Karapitiya Hospital, National Dental Hospital, Colombo South Hospital, Colombo North hospital	Principal Investigator	- Giving Information<br>- Nutritional care<br>- Psychological support<br>- mindfulness therapy<br>- Coordinating of the financial allowance
Session two	1 week after being discharged from the hospital	At the house of the patient	Public Health Nursing Officer	- Address acute issues<br>- Address the functional issues<br>- Nutritional care<br>- Mindfulness therapy<br>- Psychological support<br>- Coordinating the financial allowance
Session three	3 weeks after being discharged from the hospital	At the house of the patient	Public Health Nursing Officer	- Address acute issues<br>- Address the functional issues<br>- Nutritional care<br>- Mindfulness therapy<br>- Psychological support

Presentation of the flow of the intervention along the treatment process

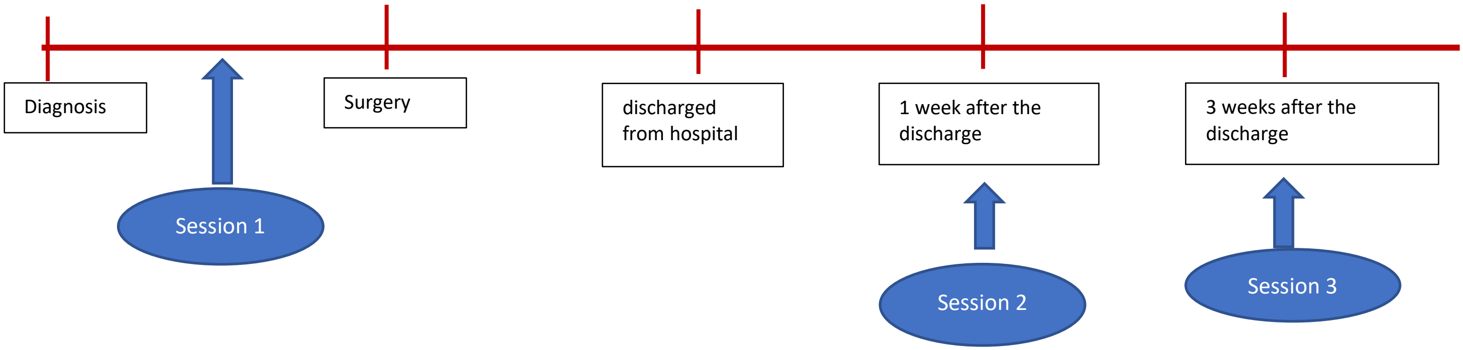

✓ **Components of the Intervention**

1. Giving Information

1st session	- A booklet will be given to the patient at the 1st visit (this booklet has been given to the PHNOs already during the training programme. ‘සෑම අඳුරු වළාවකටම රිදී රේඛාවක් ඇත..හැම අඳුරකම එළියක් ඇත’).<br>- Furthermore, a video clip will be shown to the patient and the care givers at the 1st session. This video clip will be given to the PHNOs as well to be used when needed.<br>- This first session will be carried out by the PI
2nd & 3rd sessions	- The PHNO could give the needed information when the patient has any problem. Please take extra care to give the correct information in a positive manner

2. Nutritional care

1st session	- advice on maintaining proper nutrition after surgery will be provided by the PI.
At the discharge	- a diet plan will be provided to the patient in consultation with the nutritionist.
2nd & 3rd sessions	- Check patient's compliance for the diet plan<br>- Carry out needed diet counselling<br>- If severe defect in nutrition is identified note it down on the patient's record card (annex 1) and refer to the relevant nutritionist.<br>▪ MO Nutritionist, Apeksha Hospital<br>▪ MO Nutritionist, Karapitiya

3. <u>Mindfulness Therapy</u>

1st session	- Explain the relationship between the mind and the body eg. When one gets angry the whole body reacts.<br>- Explain the advantages of being fully present in the current moment for a patient with cancer.<br>- Carry out one simple (5 mins) session focusing on breathing<br>- Share the experience and difficulties faced while doing the session.<br>- Ask patient to practice at home the following<br>▪ Carrying out the routine work in fullest concentration<br>▪ Daily sessions at least for 5 mins per day focusing on breathing
2nd session	- Ask for patient's experience, failed attempts etc.<br>- If patient hasn't successfully practiced mindfulness then probe into the failures and help the patient to re start.<br>- If the patient has followed the instructions properly then move on to the next level. Introduce mindful sitting and mindful walking. Ask the patient to practice this 10 mins per day.
3rd session	- Ask for patient's experience, failed attempts etc.<br>- If patient hasn't successfully practiced mindfulness then probe into the failures and help the patient to re start.<br><br>- If the patient has followed the instructions properly then move on to the next level. Train for being mindful about feelings. Also introduce mindful eating as well. Instruct patient to practice this 20 mins daily.

4. Coordinating of the financial allowance

1st Session	- The patient and the care givers will be informed about the financial allowance which is already available at the District Secretariat (DS) of the area.<br>- The documents that are needed from the hospital (i.e the form certifying the medical condition, the diagnosis card) will be provided to the patient.<br>- The patient will be advised to,<br>• visit the DS on a Wednesday and submit the above documents to the Grama Sewa Niladari and the Social worker.<br>• Fill the relevant form which will be provided by the Social Worker and submit to the same social worker.
2nd Session	- Find out the status of the financial allowance<br>- If any issue is identified try to rectify by contacting the relevant officers.

5. Addressing acute issues

2nd & 3rd sessions	- If there are any complains of pain from the patient this will be assessed using the pain scale (annex III).<br>- Reduce the emotional aspect of pain through counselling.<br>- Based on the pain scale if referral is needed it carry it out using the referral card (Annex II).<br>- The surgical wound site will be assessed for any acute issues like infections. If any problem is identified the needed referrals should be done through the referral card.

6. <u>Address the functional issues</u>

3rd session	- ask for any difficulties in the following functions from the patient.<br>▪ difficulties in eating<br>▪ difficulties in swallowing<br>▪ difficulties in speech<br>▪ Saliva drooling<br>▪ halitosis<br>▪ Esthetic concerns<br>-Provide help to the patient and caregivers accordingly.<br>▪ educate the patient and the caregiver regarding the specific issue (i.e. the reason for the issue, how long it will last)<br>▪ offer the remedies to lessen the problem.<br>▪ explain on the treatments available (eg. Methods of speech rehabilitation)<br>▪ If needed refer the patient to health professionals for further management.

7. <u>Psychological support</u>

This will help the patient and the family members to,

- Overcome the distress caused by the disease itself
- To manage social and relationship issues

2nd & 3rd sessions	- Listen to the patient<br>- Ventilation of emotions – let the patient express his/her emotions freely.<br>- Information and education<br>- Maintaining hope<br>- Problem solving counseling

- ✓ **Administrative procedure**

-The official permission has been already taken from the Deputy Director General Public Health Services II and Deputy Director General Non Communicable Diseases to involve the PHNOs for the intervention.

- Furthermore, when a Public Health Nursing Officer is required to visit a patient with oral cancer at his residence the relevant Head of the Institution will be officially informed.

- ✓ **Facilitation for the intervention**

-The principal investigator will be contactable always when the PHNO is in need of any technical advice or logistics issues. This will support the smooth flow of the intervention.

-A transport allowance will be provided to the Public Health Nursing Officer to facilitate visiting of the patients.

-The consultants of the tertiary care institutes will be informed about the novel intervention. Especially they will be informed regarding the referral Card.

- ✓ **'Must Do's**

-The whole purpose of this intervention is to lessen patients' psychological distress. This will be a sensitive event. Therefore, always give priority to patients need.

-The guidelines given here will only provide you the basic framework to work on. Please be sensitive to each patient and provide a tailor-made intervention for each patient.

- When there is any doubt or challenge the Principal Investigator should be contacted (Dr. Nadisha Ratnasekera – 0773752968)

-Please refer to the notes and books received at the training programme at all times. Kindly remember that although you went through two training programmes the delivery of the intervention partially depends on your attitude

- Throughout the intervention the care givers should be included.
- All the procedures done should be entered to the patient record card (Annexure I)
- The patient record card should be given to the patient and explain the importance of it.
- Whenever the referrals are been done use the referral card (Annex II)

Annex I

Patients 'record form for the intervention package – *Palliative Care Study*

Name of the patient- …………………………..

Session no. (Date)	Giving Information	Nutritional care	mindfulness therapy	Coordinating of the financial allowance	Address acute issues	Address the functional issues	Psychological support	Name & Signature of the officer who carried out the intervention
1								
At the discharge								
2								
3								

Special Note - ………………………

Annex II

Palliative care- Oral Cancer patients

Referral Card

………………………………………………………..

………………………………………………………….

…………………………………………………………

Dear Sir/ Madam,

Name of the patient –…………………………………………………

Age - ……………………………………………………

Diagnosed condition – ……………………………………………………..

Date of referral - …………………………………………………………

Reasons for the referral - ………………………………………………………………………

I would be very much grateful if you could please see to this patient and do the needful.

Thank you.

……(sign.)……………………………………..
Public Health Nursing Officer
