# Supplementary material for: Early palliative care for patients with oral cancer in Sri Lanka: A non-randomized controlled trial
Source: PLOS Glob Public Health. 2026 Mar 9;6(3):e0005985. doi: 10.1371/journal.pgph.0005985 (PMC12970869; doi:10.1371/journal.pgph.0005985)
Supplement: S4 Appendix — (DOCX) [file pgph.0005985.s004.docx]

**Process Indicators comparing the reception* of early palliative care between the intervention and control groups**

| **Intervention package components** | **Process indicator** | **Results** | | **Significance** |
| --- | --- | --- | --- | --- |
|  |  | **Intervention Group (N=55)**  **%(n)** | **Control Group (N=55)**  **%(n)** |  |
| **Providing information** | Patients who were aware of the basic information of the cancer journey | 74.5% (41) | 63.6% (35) | p= 0.001 |
| **Nutritional care** | Patients who were following a diet plan | 69.1% (38) | 16.4% (9) | p= 0.02 |
| **Coordinating of the financial allowance** | Patients who received the financial allowance | 70.1% (39) | 34.5% (19) | p= 0.835 |
| **Addressing acute and functional issues** | Patients who had their pain managed | 92.3% (4) | 67.3% (18) | p= 0.01 |
| **Psychological support and mindfulness therapy** | Patients who followed any type of mindful-based practice daily | 78.2% (43) | 47.2% (26) | p= 0.001 |

*We operationalised reception as how well participants accepted and adhered to the intervention

**The questions asked by the PI from the participants at their final follow up visit.**

1. Do you know the treatment options you have? Yes/ No

(If ‘yes’ clarified their knowledge by asking them to name the treatment options)

**2.** Have you been able to follow the diet plan provided by the hospital? Yes/ No

(Even if the participant followed the diet plan to some extent, it was considered as ‘yes’)

**3.** Did you receive the financial allowance without hazel?Yes/ No
**4.** Did you find that this new intervention was able to address and manage your pain? Yes/ No
**5.** Did you daily practice any mindfulness activity that was suggested by the Public Health Nursing Officer? Yes/ No
