# Supplementary material for: Early palliative care for patients with oral cancer in Sri Lanka: A non-randomized controlled trial
Source: PLOS Glob Public Health. 2026 Mar 9;6(3):e0005985. doi: 10.1371/journal.pgph.0005985 (PMC12970869; doi:10.1371/journal.pgph.0005985)
Supplement: S5 Appendix — (DOCX) [file pgph.0005985.s005.docx]

**Impact of COVID-19 to the study**

Due to the COVID-19 threat, there was a slight deviation in the timings of the intervention package delivery (sessions 2 & 3) and data collection of both intervention and control groups due to the following valid reasons.

- - - Recruitment of Public Health Nursing Officers, for COVID-19 prevention programmes which was needed at the time. Since Public Health Nursing Officers were supposed to carry out the last two sessions of the intervention, there was a delay in delivering the sessions.
    - The stringent COVID-19 lockdown period prevailed in the country since 20^th^ March 2020 which went on for one month with a near absence of public transport and permitted limited private transport made visiting the patients at their residences challenging for Public Health Nursing Officers.
    - Due to the reorientation of health care exclusively for emergency visits, postponing the scheduled treatments/follow-up clinics for patients with cancer was evident during this period. This affected the post-intervention data collection of both intervention and control groups.

We carried out a post hoc analysis among the participants whose timings were affected by COVID-19 and the participants who were non-affected in the intervention and control groups to find out whether the delays had any bias in assessing the effectiveness of the intervention. The operationalized definition of the COVID-19 affected participants was as follows;

* COVID-19–affected participants – Participants whose scheduled timing for the delivery of the intervention sessions, standard care, or follow-up data collection was delayed or rescheduled due to restrictions or disruptions related to the COVID-19 pandemic (e.g., unavailability of Public Health Nursing Officers for home visits, or inability of participants to attend hospital clinic visits because of lockdowns or travel restrictions).

** COVID-19–unaffected participants – Participants whose intervention delivery, standard care, and data collection occurred according to the planned schedule and were not delayed or disrupted by the COVID-19 pandemic.

Table (i) compares the Distress Thermometer scores at 3 months post-intervention (T_3_) between the COVID-19-affected and unaffected participants in the intervention group.

Table (i): Comparison of the Distress Thermometer scores at 3 months post-intervention (T_3_) between the COVID-19 affected and unaffected participants in the intervention group

|  | **Unaffected by COVID-19^b^**  **(n= 43)** | | **Affected by COVID-19^a^ (n=12)** | | **P value*** |
| --- | --- | --- | --- | --- | --- |
|  | **Mean (SD)** | **Median (IQR)** | **Mean (SD)** | **Media (IQR)** |  |
| DT score at T_3_ | 3.47 (2.7) | 3 (2.0) | 3.75 (3.3) | 3.0 (5.5) | 0.94 |

DT – Distress Thermometer

T_3_- Three months post-intervention

a= Any study participant whose correct timing of the novel intervention package, the standard care, or data collection got affected due to COVID-19 pandemic

b= Any study participant whose correct timing of the novel intervention package, the standard care or data collection did not get affected due to COVID-19 pandemic

* Mann Whitney U-test

According to table i, there is no significant difference among the DT scores of the COVID-19 affected and unaffected groups.

Table (ii) presents the comparison of the Distress Thermometer scores at 3 months post-intervention T_3_ between the COVID-19 affected and unaffected participants in the control group

Table (ii) Comparison of the Distress Thermometer scores at 3 months post-intervention (T_3_) between the COVID-19 affected and unaffected participants in the intervention group

|  | **Unaffected by COVID-19^b^**  **(n= 50)** | | **Affected by COVID-19^a^ (n=5)** | | **p value*** |
| --- | --- | --- | --- | --- | --- |
|  | **Mean (SD)** | **Median (IQR)** | **Mean (SD)** | **Media (IQR)** |  |
| DT score atT_3_ | 5.7 (2.9) | 6 (4.2) | 4.6 (3.0) | 6 (5.5) | 0.52 |

DT – Distress Thermometer

T_3_- 3 months post-intervention

a= Any study participant whose correct timing of the novel intervention package, the standard care, or data collection was affected due to COVID-19 pandemic

b= Any study participant whose correct timing of the novel intervention package, the standard care or data collection did not get affected due to COVID-19 pandemic

* Mann Whitney U-test

As per table (ii) the Distress Thermometer scores of COVID-19 affected and unaffected groups do not show a significant difference. Therefore, delays caused by COVID-19 have not affected the effectiveness of the novel intervention package.
